# A novel approach to modeling epidemic vulnerability, applied to *Aedes aegypti*-vectored diseases in Perú

**DOI:** 10.1186/s12879-021-06530-9

**Published:** 2021-08-21

**Authors:** Julianne Meisner, Lauren A. Frisbie, César V. Munayco, Patricia J. García, César P. Cárcamo, Cory W. Morin, David M. Pigott, Peter M. Rabinowitz

**Affiliations:** 1 Department of Epidemiology, University of Washington, Seattle, WA USA; 2 Center for One Health Research, Department of Environmental and Occupational Health Sciences, University of Washington, Seattle, WA USA; 3 Centro Nacional de Epidemiología, Prevención y Control de Enfermedades, Peruvian Ministry of Health, Lima, Peru; 4 School of Public Health and Administration, Universidad Peruana Cayetano Heredia, Lima, Peru; 5 Center for Health and the Global Environment, Department of Environmental and Occupational Health Sciences, University of Washington, Seattle, WA USA; 6 Institute for Health Metrics and Evaluation, University of Washington, Seattle, WA USA

**Keywords:** Outbreak, Epidemic, Spatial epidemiology, Vectorborne diseases, Perú, Dengue

## Abstract

**Background:**

A proactive approach to preventing and responding to emerging infectious diseases is critical to global health security. We present a three-stage approach to modeling the spatial distribution of outbreak vulnerability to *Aedes aegypti*-vectored diseases in Perú.

**Methods:**

Extending a framework developed for modeling hemorrhagic fever vulnerability in Africa, we modeled outbreak vulnerability in three stages: index case potential (stage 1), outbreak receptivity (stage 2), and epidemic potential (stage 3), stratifying scores on season and El Niño events. Subsequently, we evaluated the validity of these scores using dengue surveillance data and spatial models.

**Results:**

We found high validity for stage 1 and 2 scores, but not stage 3 scores. Vulnerability was highest in Selva Baja and Costa, and in summer and during El Niño events, with index case potential (stage 1) being high in both regions but outbreak receptivity (stage 2) being generally high in Selva Baja only.

**Conclusions:**

Stage 1 and 2 scores are well-suited to predicting outbreaks of *Ae. aegypti*-vectored diseases in this setting, however stage 3 scores appear better suited to diseases with direct human-to-human transmission. To prevent outbreaks, measures to detect index cases should be targeted to both Selva Baja and Costa, while Selva Baja should be prioritized for healthcare system strengthening. Successful extension of this framework from hemorrhagic fevers in Africa to an arbovirus in Latin America indicates its broad utility for outbreak and pandemic preparedness and response activities.

**Supplementary Information:**

The online version contains supplementary material available at 10.1186/s12879-021-06530-9.

## Background

As made clear by the COVID-19 pandemic—as well as the Zika virus and Ebola virus epidemics of the mid 2010s, and the MERS and SARS coronavirus outbreaks that preceded them—emerging infectious diseases threaten global economies, population health, and global health security. A proactive approach to such diseases, in particular tools to facilitates health-system strengthening while optimizing limited resources, is needed to close gaps in pandemic preparedness. To this end, a collaboration between the University of Washington’s MetaCenter for Pandemic Preparedness and Global Health Security and the Universidad Peruana Cayetano Heredia has developed a suite of mapping tools to characterize, in Perú, district-level vulnerability to outbreaks of *Aedes* spp.-transmitted viruses. We have used surveillance data for dengue, the most frequently reported cause of acute febrile illness in Latin America [1], to demonstrate the validity of these tools.

Transmitted by *Ae. aegypti*, dengue is caused by four closely related dengue virus serotypes: DENV-1, DENV-2, DENV-3, DENV-4. While a continental effort to eradicate *Ae. aegypti* in the mid-20th century succeeded in Perú [2], re-infestation in 1984 was followed by re-emergence of dengue in 1990 [3]. Numerous major dengue epidemics have occurred since, and Perú is now considered one of the 30 most highly endemic dengue infection countries in the world, with an average of almost 10,000 cases reported to WHO between 2004 and 2010 and over 56,000 cases reported in 2020 [4–6].

El Niño Southern Oscillation (ENSO) is a periodic phenomenon which drives variability in rainfall patterns and temperature, and increases the likelihood of extreme weather events [7]. In 2017, following an ENSO event, Perú experienced its largest dengue epidemic to date: between February and July over 65,000 cases were reported, representing a three-fold increase over the same period in 2016. Nearly 90% of these cases reported from four coastal departments particularly affected by the event [8, 9].

This study builds on a framework previously applied to hemorrhagic fevers in Africa, which stratifies vulnerability on three key transition points in a potential epidemic. Stage 1 reflects potential for occurrence of an index case, modeled using weather and population distribution data. Stage 2 captures potential for a localized outbreak, modeled using measures of healthcare system strength and access. Stage 3 reflects potential for a widespread epidemic, modeled using distance to population centers, regional and national borders, and airports [10]. While prior authors have leveraged vector habitat suitability data and epidemiologic data to estimate sub-national spatiotemporal risk for Zika virus transmission [11, 12], climate indicators to model dengue risk [13], and meteorological variables to simulate dengue outbreaks in a dynamic modelling framework [14], other drivers of dengue outbreaks are less commonly utilized for risk prediction. Furthermore, this study is the first attempt to disaggregate risk by stage, with implications for intervention design.

Stratifying on season and El Niño, and using surveillance data and a spatially-explicit approach, we sought to identify: (1) which districts in Perú are most vulnerable to dengue outbreaks, (2) the extent to which each stage of vulnerability leads to dengue outbreaks, and (3) the predictive ability of each vulnerability stage for dengue outbreaks.

## Methods

The STROBE checklist was used to guide drafting of this manuscript [15].

### Setting

The study area includes all 1851 districts in Perú. Data on dengue cases (outcome) were provided by Perú’s national dengue surveillance system, coordinated by the Ministerio de Salud. These data were collected from January 2016 to September 2018. The study population includes all individuals under surveillance for dengue in Perú.

**Fig. 1:**
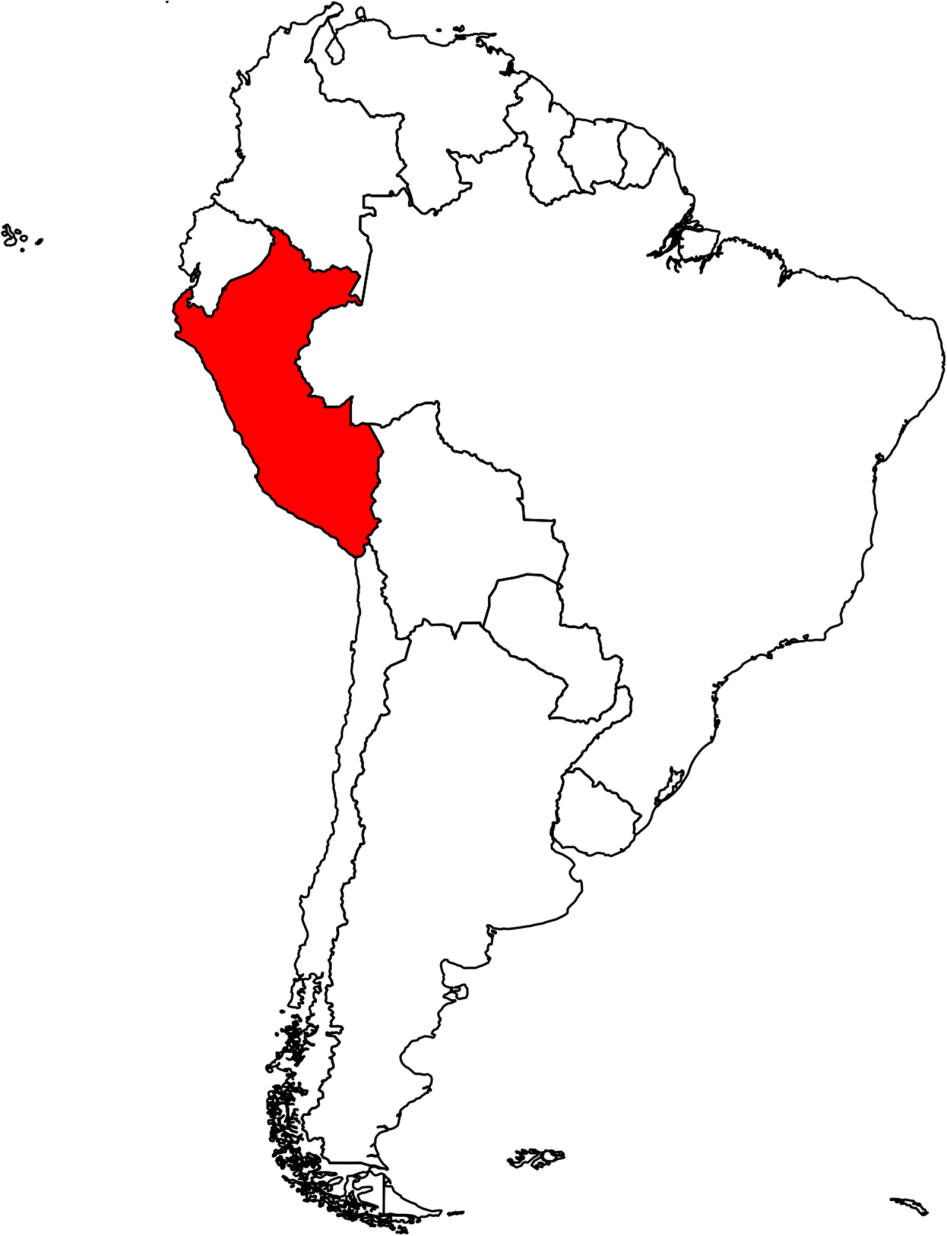
Location of Perú within South America

**Fig. 2:**
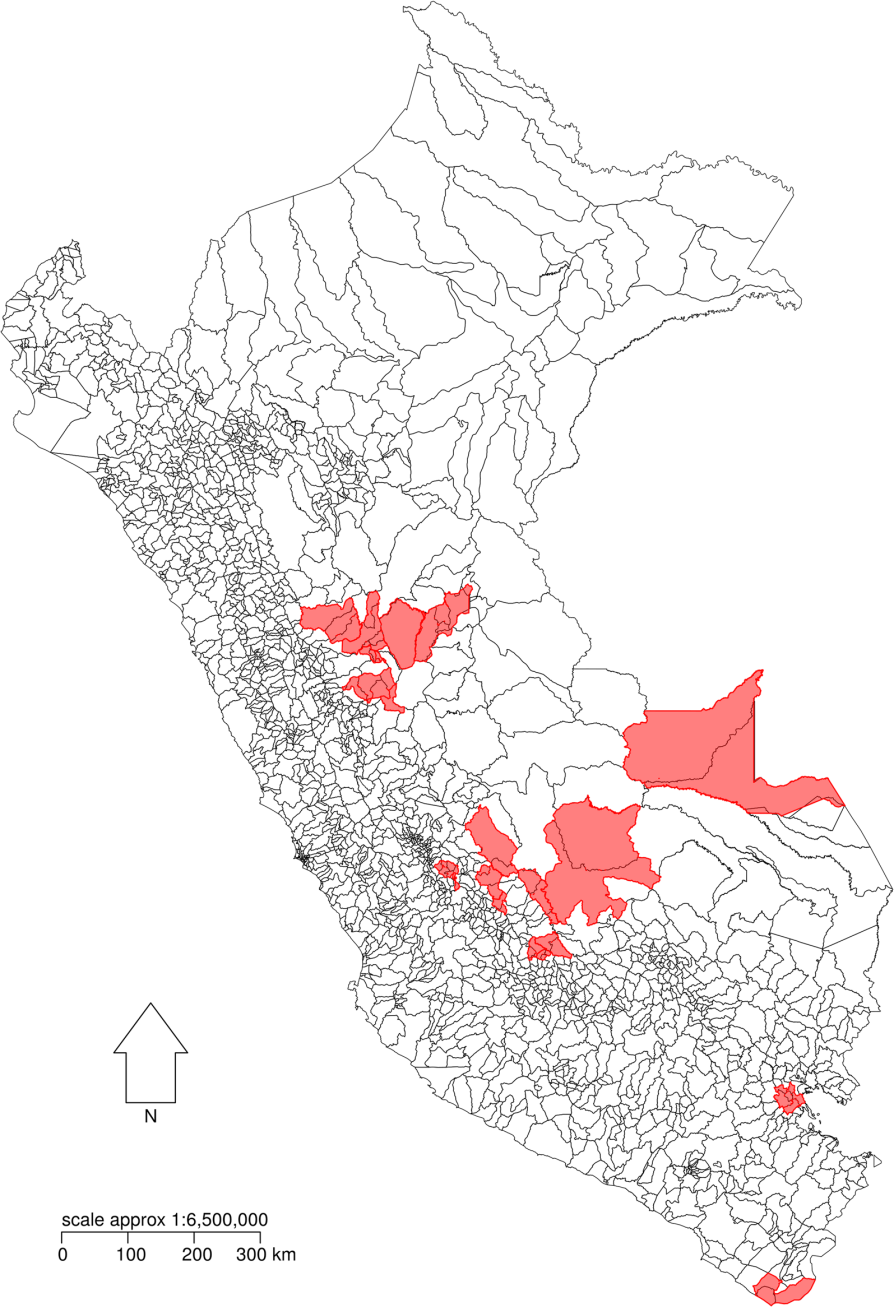
Perú. Study districts are outlined in black, and merged districts are shaded in red. The merged districts were created by collapsing districts formed during the study period with their “parent” district(s)

As some districts were formed during the study period, and novel districts would not be contributing to reporting prior to their inception, for inference and prediction we merged newly-formed districts with their parent districts to eliminate missingness in the outcome variable, generating a total of 1838 districts for analysis (Figs. 1 and 2). In doing this we took the mean of the vulnerability scores calculated for each parent district (detailed below) as the merged district’s score, separately for each of the three stages.

### Vulnerability mapping

*Stage 1* To determine risk of an index case of dengue, we used a weather-based model of vector incubation and survival adjusted for human population. Vector incubation and survival was calculated using temperature data .

We obtained surface air temperature data using NASA’s Global Land Data Assimilation System (GLDAS) [16]. GLDAS uses both observed and modeled meteorological data to force land surface models. We used data from the Noah-LSM version 2.1 with a spatial resolution of 0.25$^{\circ }$ $\times$ 0.25$^{\circ }$ and a temporal resolution of 1 month. From these data we calculated the mean temperature for summer (November–April) and winter (May-October) across the study period. Additionally, we calculated the average temperatures for each season during El Niño vs non-El Niño years. This was done by first calculating the average MEI (Multivariate ENSO index) for each season (winter vs summer as described above) and year from 1950–2017, then selecting the upper quantile years within the study period as El Niño years and the remaining years during the study period as non-El Niño years. We then calculated the average temperature for both the El Niño years and non-El Niño years by season.

The temperature data were then used to calculate the probability of an *Ae. aegypti* mosquito surviving the extrinsic incubation period (EIP). The EIP is the period between when a mosquito takes an infectious blood meal and when it can transmit the virus through a subsequent feeding. We assumed a lower limit for transmission of 0$^{\circ }$C and a constant adult daily mosquito mortality probability of 0.86 [17, 18]. The equation for the length of the EIP as a function of temperature was derived from data collected by Tjaden et al. (2013) [19]:

$$\begin{aligned} EIP(T) = 1.0033 e^{-0.077T} \end{aligned}$$

where EIP is the extrinsic incubation period and T is temperature. Using this equation and the estimated daily survival probability, we estimated the probability that an *Ae. aegypti* mosquito will survive the EIP at a given temperature:

$$\begin{aligned} SEIP(T) = 0.86^{EIP(T)} \end{aligned}$$

where SEIP(T) is the probability of survival past the EIP and T is temperature.

In order to assign a value to each district we took the mean of the gridded temperature and SEIP that fell within each district. Because this was based on the center of the cell, some of the small districts did not contain the center of a grid cell. To account for this, we made a 12.5km buffer around these districts and then took the mean of those cells whose center fell within them. This produced both a mean temperature and mean SEIP for each season (summer and winter) and for each season during El Niño and non-El Niño years.

Human population data for 2016 was obtained from WorldPop [20]. Population data and vector index were combined to produce a relative stage 1 (index case) vulnerability score for each district.

*Stage 2* To calculate stage 2 vulnerability (outbreak receptivity), we combined stage 1 vulnerability scores for each district with a proxy score for health system capacity to contain an outbreak, derived from vaccine coverage, under 5 mortality estimates, and travel time to healthcare facilities.

Estimates of DPT vaccine coverage for 2018 were obtained from the Ministry of Health of Perú (MINSA) [21]. Age-specific proportion unvaccinated was standardized within each age group, then the arithmetic mean taken over these groupings. Under 5 mortality estimates for 2017 were obtained from Burstein et al. [22], and travel time to the nearest health facility was estimated using a global friction layer from Weiss et al. [23]. Health facilities were discretized into three tiers defined by the Perúvian government (public facilities, social insurance system, and private facilities), and the arithmetic mean taken over these tiers [24]. The geometric mean was taken over these input values to generate outbreak receptivity scores.

*Stage 3* For stage 3 (epidemic potential), we combined stage 2 information with calculated travel time to the nearest city of 50,000 persons or more, derived from Weiss et al [23].

All scores were standardized to a 0–10 scale, separately for each climate scenario, using a Box-Cox transformation to normalize their distribution.

### Regression models

*Study design* This study was an ecological retrospective cohort study, conducted at the district level. All districts in the study period were included, thus no sample size calculations were performed.

*Exposure* Exposure was stage-specific vulnerability score, as detailed above.

*Outcome* Variables extracted from the dengue surveillance data include district of residence, date of initial symptoms, and diagnosis type (probable, confirmed, and discarded). We collapsed probable and confirmed cases for all analyses, where probable cases were defined by history of recent fever in addition to two or more symptoms, and confirmed cases additionally had either (1) positive serum isolation, (2) four-fold change in IgM or IgG titer, (3) positive PCR, (4) positive immunoassay, or (5) an epidemiological link [21].

To mitigate surveillance fatigue as a driver of misclassification, we parameterized outcome as dengue outbreaks. An outbreak was defined as five or more cases with symptom onset within three or more consecutive weeks, extending forward and backward in time until at least two weeks with no reported cases occurred [25].

We then collapsed in time, estimating the (a) total number of outbreak weeks, (b) whether one or more outbreaks occurred, and (c) median duration of outbreaks in each district over the study period.

*Confounders* As vulnerability is both broadly-defined and latent, we could conceive of no variables that could be confounders (i.e., causes of dengue outbreaks which are associated with but not downstream of vulnerability) that would not themselves be considered a component of vulnerability. Thus, our inferential models included no confounders.

**Fig. 3:**
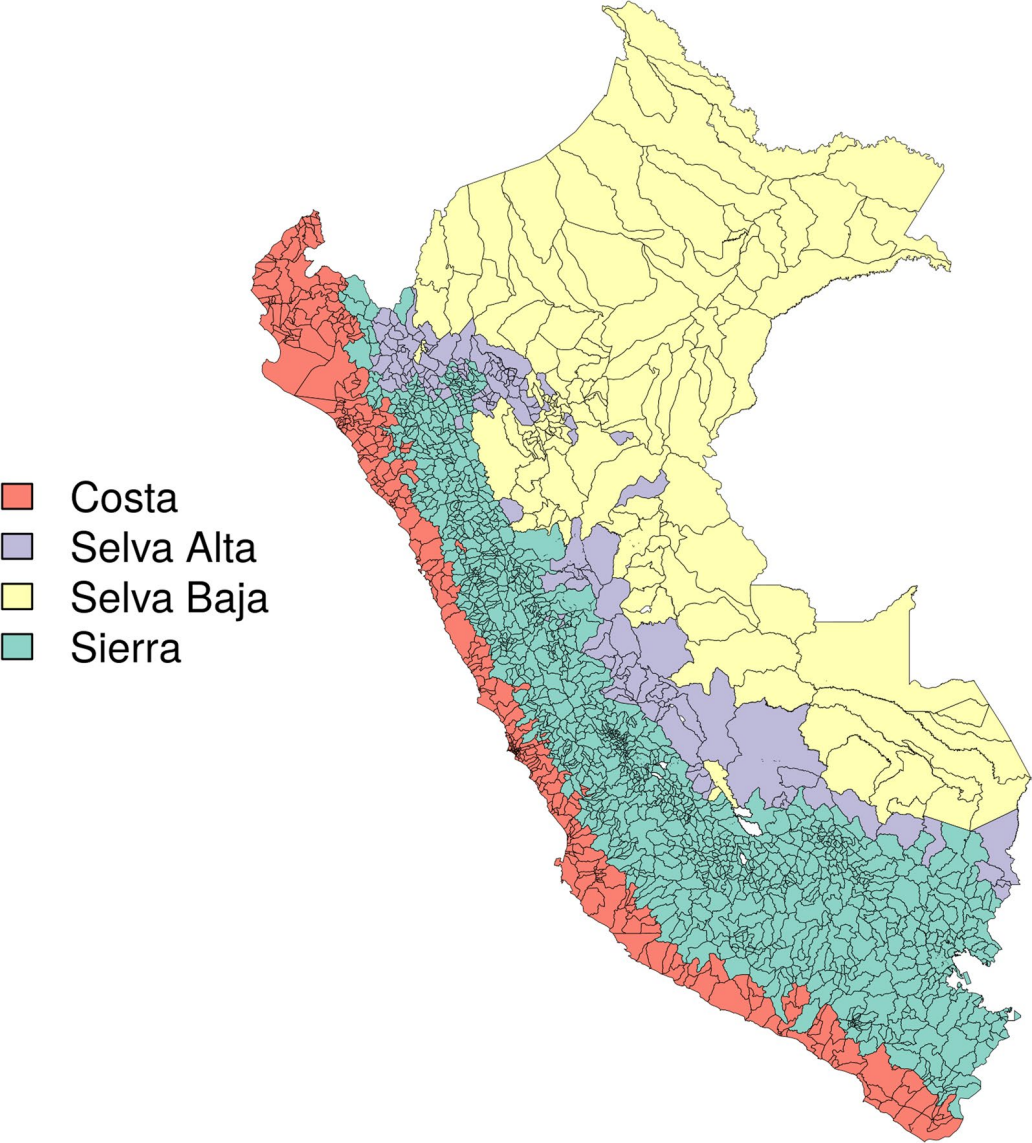
Natural regions over merged districts in Perú

*Effect modifiers* As connectivity may have differential effects on outbreak risk in different eco-regions, in a sensitivity analysis we modeled stage 3 vulnerability with an interaction term for natural region (Selva Alta, Sierra, Selva Baja, Costa; Fig. 3). Natural region data were downloaded from a Universidad de San Martín de Porres database [26].

We also subset the data into summer versus winter, El Niño versus non-El Niño, and all combinations thereof, as vulnerability scores are specific to binary season and El Niño activity as detailed above. We defined summer as December to April, winter as May to November, and El Niño using monthly El Niño Coastal Index (ICEN) data. This index was developed by Perú’s Estudio Nacional del Fenómeno El Niño and reflects local ENSO impacts. We downloaded these data from the Instituto del Mar del Perú website [27]. Months with a sea surface temperature anomaly of greater than 0.4$^{\circ }$C were classified as El Niño.

*Statistical analyses* We fit all models (inference and prediction) as Bayesian hierarchical spatial smoothing models, using the R-INLA package. District-level random effects $e_i$ included both structured (spatial) and unstructured (non-spatial) components:

1 $$\begin{aligned}&e_i = S_i + \epsilon _i \end{aligned}$$

2 $$\begin{aligned}&\epsilon _i|\sigma _\epsilon ^2 \sim {_{iid} N(0, \sigma _\epsilon ^2)} \end{aligned}$$

3 $$\begin{aligned}&\mathbf{S} |\sigma _s^2 \sim {ICAR(\sigma _s^2)} \end{aligned}$$

where

- $\epsilon _i$ are the unstructured (non-spatial) random effects
- $\mathbf{S}$ is the vector of structured (spatial) random effects.
- and ICAR is the intrinsic conditional autoregressive model, in which each district’s random effect is a function of its neighbors’

For both random effects we used a “penalized complexity” prior [28, 29] $Pr(\sigma >U)=\alpha$, where $\sigma$ is the standard deviation for the structured and unstructured random effects, $U=1$, and $\alpha =0.01$. With a log link, this specification gives a 99% posterior credible interval of (0.31, 2.72), on the multiplicative scale, for each random effect’s residual relative risk.

*Inference* For inference, we fit two families of models: zero inflated Poisson models with outcome parameterized as total outbreak weeks, and logistic regression models with outcome parameterized as binary presence versus absence of an outbreak week observed over the study period.

In a sensitivity analysis, we fit a zero inflated Poisson model with outcome parameterized as median outbreak duration to the stage 2 vulnerability score, as healthcare system strength may have a greater impact on duration of an outbreak than presence of an outbreak.

*Prediction* For prediction, we set outcome to missing for a random one-third of districts in our dataset. This allows these districts to serve as a test set, while ensuring their random effects will still be estimated (required for prediction from a spatial model), compared with removing these districts from our data entirely. We then fit logistic regression models, with binary outcome as described above, to the “training” data. We produced ROC curves and estimated AUC for each district using the ROCR package in R.

## Results

### Vulnerability mapping

**Fig. 4:**
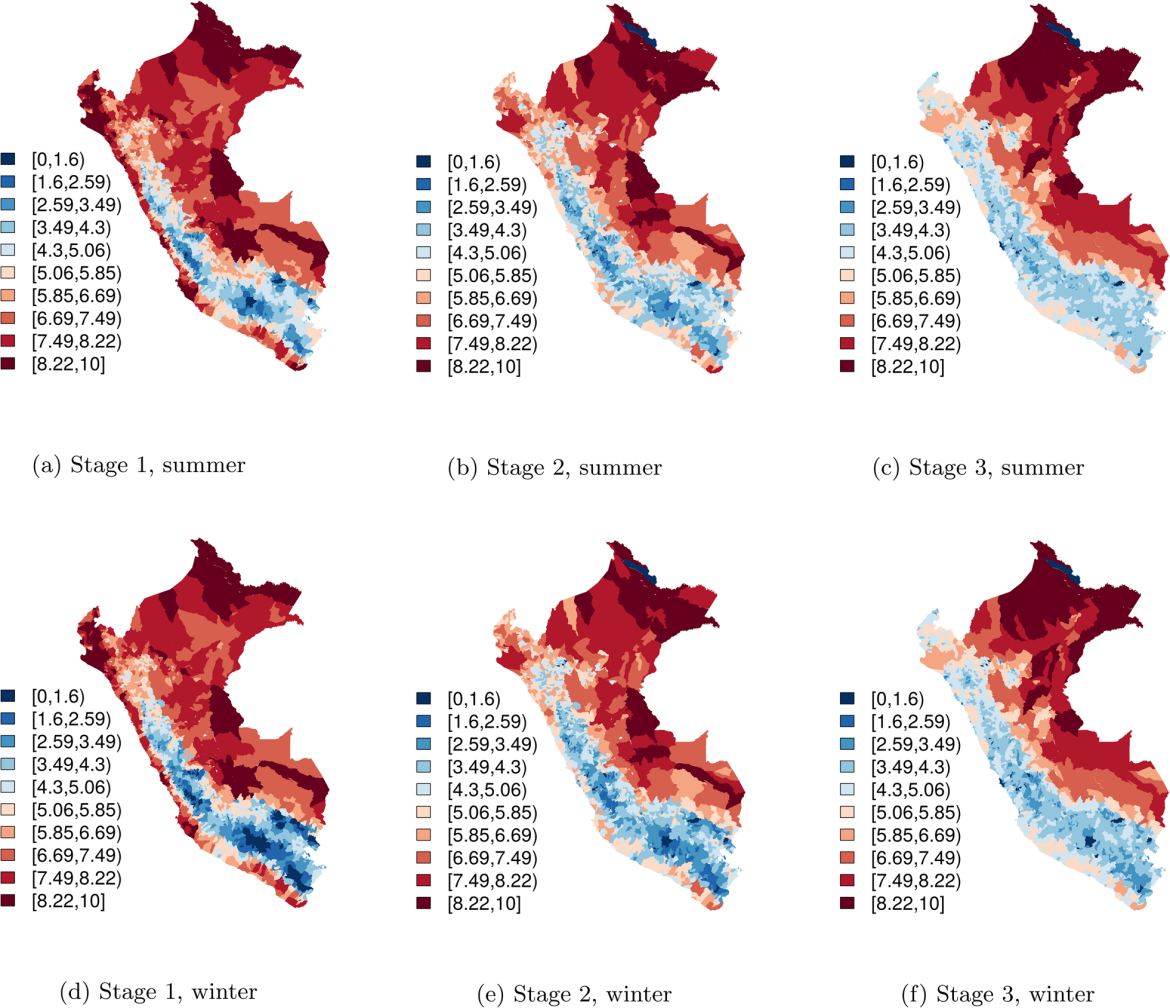
Vulnerability scores for summer (November–April) and winter (May–October), mean over the study period

Vulnerability maps for stages 1, 2, and 3 by season are presented in Fig. 4. The same maps stratified on El Niño and non-El Niño period are presented in additional figures [see Additional files 1 and 2], as well as a hyperlink to an interactive version [see Additional file 3]. Across all three stages, vulnerability was lowest in the highlands (Selva Alta and Sierra). Stage 1 vulnerability (index case potential) was highest in the Selva Baja and Costa ecozones, while stage 2 (outbreak receptivity) was in general highest in Selva Baja, and stage 3 (epidemic potential) was again highest in the Selva Baja ecozone, however with more high-risk districts in the northern extent of this ecozone than the southern extent.

Descriptive statistics for vulnerability scores are presented in Table 1; these are parameterized to range from 0 to 10.

**Table 1:** Descriptive statistics, vulnerability scores

Stage	Season	El Niño	Mean	SD
Stage 1	W	−	5.12	1.96
	S	−	5.46	1.73
	W	N	5.15	1.93
	W	Y	5.48	1.72
	S	N	5.20	1.89
	S	Y	5.50	1.69
Stage 2	W	−	4.44	1.49
	S	−	4.62	1.39
	W	N	4.46	1.47
	W	Y	4.63	1.38
	S	N	4.49	1.45
	S	Y	4.64	1.38
Stage 3	W	−	4.16	1.37
	S	−	4.26	1.33
	W	N	4.18	1.36
	W	Y	4.27	1.32
	S	N	4.19	1.35
	S	Y	4.27	1.32

Vulnerability score by stage and El Niño. Stage 1: index case potential; stage 2: outbreak receptivity; stage 3: epidemic potential. W: winter; S: summer; N: no; Y: yes

### Regression models

**Fig. 5:**
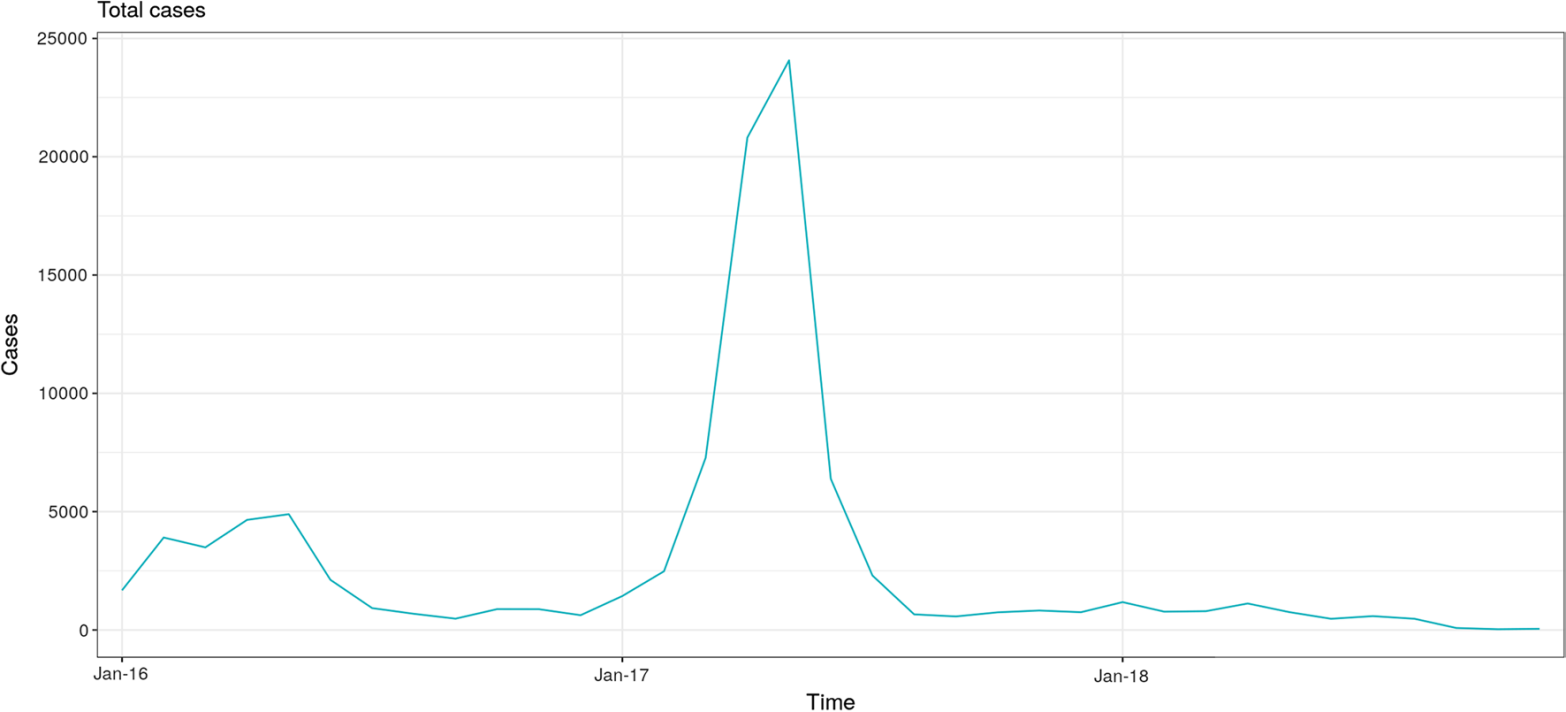
Time series of dengue cases in all districts of Perú, January 2016−September 2018

**Fig. 6:**
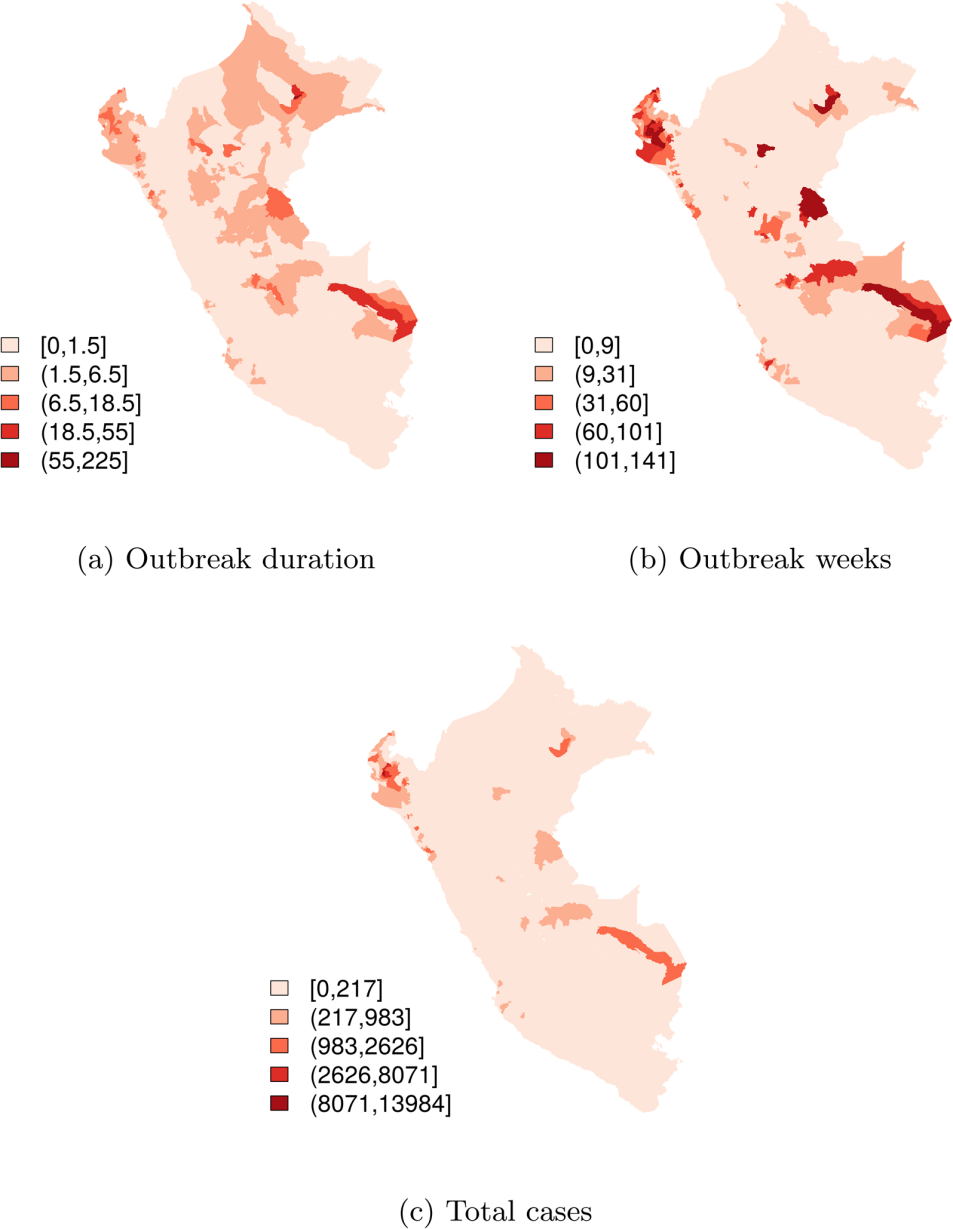
Median duration of outbreaks, total number of outbreak weeks, and total number of cases, reported by each district from January 2016−September 2018

*Descriptive statistics* A total of 99,789 dengue cases were reported during the study period, with a peak of cases observed in 2017 (Fig. 5). Out of 1838 districts, 174 (9.5%) experienced at least one dengue outbreak during the study period. The mean number of outbreaks experienced was 0.79 (range 0, 27), mean number of outbreak weeks was 3.62 (range 0, 141), and mean outbreak duration was 0.68 weeks (range 0, 225) (Fig. 6).

*Inference* Results from inferential models are presented in Tables 2 (zero-inflated Poisson model) and 3 (logistic regression model). Point estimates were strong for both models, ranging from a 11% higher number of outbreak weeks (zero-inflated Poisson model) and 49% higher odds of an outbreak (logistic model) for a district with a one unit higher stage 3 vulnerability score in the overall winter and El Niño winter models, respectively; to a 28% higher number of outbreak weeks (zero-inflated Poisson model) or 456% higher odds of an outbreak (logistic model) for a district with a one unit higher vulnerability score for the stage 2 overall summer and winter (equivalent point estimates) and stage 1 summer El Niño models, respectively. There were no clear trends in rate ratios across stage, season, or El Niño (Table 2), however odds ratios decreased with increasing stage (Table 3).

**Table 2:** Zero-inflated Poisson model results

	Season	El Niño	RR (95% CI)
Stage 1	S	–	1.24 (1.20, 1.29)
	W	–	1.26 (1.21, 1.31)
	S	Y	1.24 (1.19, 1.29)
	S	N	1.15 (1.09, 1.20)
	W	Y	1.19 (1.14, 1.23)
	W	N	1.24 (1.19, 1.30)
Stage 2	S	–	1.28 (1.22, 1.34)
	W	–	1.28 (1.22, 1.34)
	S	Y	1.27 (1.21, 1.33)
	S	N	1.20 (1.13, 1.26)
	W	Y	1.21 (1.16, 1.26)
	W	N	1.26 (1.20, 1.32)
Stage 3	S	–	1.19 (1.14, 1.25)
	W	–	1.11 (1.05, 1.18)
	S	Y	1.19 (1.13, 1.24)
	S	N	1.15 (1.09, 1.22)
	W	Y	1.12 (1.07, 1.17)
	W	N	1.15 (1.09, 1.22)

Outcome parameterized as number of outbreak weeks in a given district over January 2016-September 2018. Stage 1: index case potential; stage 2: outbreak receptivity; stage 3: epidemic potential. RR: rate ratio; CI: posterior credible interval; S: summer; W: winter; Y: yes; N: no

**Table 3:** Logistic model results

	Season	El Niño	OR (95% CI)
Stage 1	S	–	5.17 (3.94, 7.22)
	W	–	5.39 (4.05, 7.67)
	S	Y	5.56 (4.18, 7.94)
	S	N	3.74 (2.68, 5.70)
	W	Y	5.19 (3.77, 7.83)
	W	N	5.33 (3.97, 7.75)
Stage 2	S	–	3.74 (3.00, 4.89)
	W	–	3.60 (2.91, 4.64)
	S	Y	3.69 (2.95, 4.82)
	S	N	2.89 (2.28, 3.87)
	W	Y	3.45 (2.72, 4.56)
	W	N	3.46 (2.78, 4.51)
Stage 3	S	–	1.57 (1.35, 1.84)
	W	–	1.62 (1.41, 1.89)
	S	Y	1.50 (1.29, 1.75)
	S	N	1.54 (1.27, 1.88)
	W	Y	1.49 (1.26, 1.79)
	W	N	1.54 (1.33, 1.80)

Outcome parameterized as one or more outbreaks in a given district over January 2016–September 2018. Stage 1: index case potential; stage 2: outbreak receptivity; stage 3: epidemic potential. *OR* odds ratio, *CI* posterior credible interval, *S* summer, *W* winter, *Y* yes, *N* no

*Sensitivity analyses* Stage 2 vulnerability (outbreak receptivity) was found to be more strongly associated with median outbreak duration than number of outbreak weeks (Table 4). No interaction was found between stage 3 vulnerability (epidemic potential) and natural region.

**Table 4:** Sensitivity analysis: stage 2 model, median outbreak duration

Season	El Niño	RR (95% CI)
Summer	–	3.10 (2.67, 3.54)
Winter	–	3.31 (1.49, 4.40)
Summer	Y	3.18 (2.76, 3.80)
Summer	N	3.06 (2.72, 3.46)
Winter	Y	4.52 (3.74, 5.62)
Winter	N	2.54 (1.72, 3.04)

Zero inflated Poisson model results for stage 2 vulnerability score (outbreak receptivity). Outcome parameterized as median duration of outbreaks in a given district over January 2016–September 2018. *RR* rate ratio, *CI* posterior credible interval, *Y* yes, *N* no

**Table 5:** Prediction model results

	Season	El Niño	AUC (95% CI)
Stage 1	S	–	0.901 (0.874, 0.929)
	W	–	0.885 (0.853, 0.918)
	S	Y	0.877 (0.842, 0.913)
	S	N	0.859 (0.795, 0.922)
	W	Y	0.914 (0.883, 0.944)
	W	N	0.927 (0.904, 0.950)
Stage 2	S	–	0.867 (0.828, 0.905)
	W	–	0.824 (0.767, 0.882)
	S	Y	0.758 (0.689, 0.827)
	S	N	0.905 (0.862, 0.949)
	W	Y	0.889 (0.851, 0.927)
	W	N	0.866 (0.822, 0.910)
Stage 3	S	–	0.673 (0.598, 0.747)
	W	–	0.608 (0.526, 0.690)
	S	Y	0.580 (0.490, 0.670)
	S	N	0.679 (0.530, 0.828)
	W	Y	0.658 (0.564, 0.753)
	W	N	0.614 (0.521, 0.707)

Prediction results from logistic regression models trained to two-thirds of the outcome data, and tested on remaining one-third. Outcome parameterized as one or more outbreaks in a given district over January 2016–September 2018. Stage 1: index case potential; stage 2: outbreak receptivity; stage 3: epidemic potential.*AUC* area under ROC curve,*CI* posterior credible interval, *S* summer; *W* winter, *Y* yes, *N* no

*Prediction* Prediction models results are presented in Table 5. Area under the ROC curve (AUC) was in general high, being the lowest for stage 3 vulnerability, summer El Niño (0.58, 95% CI 0.49, 0.67), highest for stage 1 vulnerability, winter, non El Niño (0.93, 95% CI 0.90, 0.95), and over 0.8 for 11 out of 18 model. ROC curves are presented in Additional file 4.

## Discussion

We found index case potential to be highest in eastern Selva Baja and northern and central Costa, in both winter and summer: close to one-quarter of Selva Baja and Costa districts had high index case potential (stage 1 score > 8), versus 7% of Selva Alta districts and 0% of Sierra districts. Of these Selva Baja districts, 15% also had high outbreak receptivity, while none of the Costa districts with high index case potential also had high outbreak receptivity, and several districts in Costa (Comas, San Borja, and San Juan de Miraflores, all located in Lima province) with high index case potential had very low outbreak receptivity (stage 2 scores < 3). These findings likely reflect superior healthcare capacity in Costa than Selva Baja, and differences in climate between the two regions. Perú is a highly-centralized country: resources and capacity are concentrated in the capital city of Lima and surrounding coastal cities, with lower healthcare capacity and access in Selva Baja. Furthermore, the combination of poor housing conditions, dense vegetation, and year-round high temperatures and humidity in Selva Baja facilitates arbovirus transmission in this region, while in Costa transmission wanes and healthcare systems can “recover” during dry periods. Temporally, vulnerability was slightly lower in winter and non-El Niño periods than summer and El Niño, reflecting features of the *A. aegypti* lifecycle.

Vulnerability score was strongly associated with the risk and number of dengue outbreaks, however associations were slightly stronger for index case and outbreak receptivity than epidemic potential. This finding is not surprising, and suggests stage 3 scores (epidemic potential) are more relevant to diseases with direct human-to-human transmission rather than those which are vector-mediated. Outbreak receptivity (stage 2) demonstrated stronger association with outbreak duration than outbreak occurrence, lending support to the validity of our vulnerability model and suggesting that some districts with high index case potential but low outbreak receptivity may be successful at stemming outbreaks. Our prediction models largely support the findings of our inferential models, namely stage 1 and 2 vulnerability scores (index case and outbreak receptivity, respectively) performed extremely well for predicting dengue outbreaks, however stage 3 (epidemic potential) scores performed poorly.

Our approach has several limitations. First, the validity of our vulnerability scores is compromised by any uncertainty or bias in the predictors used to model each stage. Further, we modeled the EIP based only on temperature, however this parameter varies even within a narrow temperature range, likely reflecting the effects of other determinants including host viremia, blood meal size, viral serotype, and others. We did not include these variables in our approach as they are difficult to parameterize, and we expect their effect on EIP to be markedly weaker than temperature. Finally, in validating our model we collapsed probable and confirmed dengue cases, however probable cases may actually be due to other arboviruses—including chikungunya and yellow fever, which are also transmitted by *Ae. ageypti* mosquitoes—or malaria. As our goal was to model vulnerability to any *Ae. ageypti*-vectored disease, misclassification of these other arboviruses as probable dengue does not compromise validation of our approach. While we did not intend to model vulnerability to malaria and other disease transmitted by Anophelinae mosquitoes, misclassification of malaria as probable dengue is expected to compromise apparent validity of our models—that is, make performance appear poorer than it truly is.

Despite these limitations, our approach represents a novel addition to the arbovirus modeling literature. While numerous other models have used ENSO and meteorological variables to predict or simulate dengue outbreaks, the use of other covariates is extremely limited [14, 30–37]. These efforts have largely focused on what we refer to as index case receptivity, with a single recent application including connectivity as a covariate, and, to our knowledge, no prior use of health care system quality as a covariate [38].

Beyond its novelty, our staged approach supports planning and resource allocation to prevent and mitigate outbreaks of dengue and other *Ae. aegypti*-vectored arboviruses. Our findings suggest mosquito surveillance and control, syndromic surveillance, improved ability to identify index cases—through training of healthcare workers, improved diagnostic capacity, and other measures—and vaccination campaigns should be targeted to districts in Selva Baja and Costa with high index case potential (stage 1). Investments in health-systems strengthening, in particular outbreak preparedness and response capacities, should instead be targeted to Selva Baja districts with high outbreak receptivity (stage 2). Had we detected a stronger predictive ability for epidemic potential with dengue outbreaks, spatial distribution of epidemic potential (stage 3) could be used to target efforts for halting regional and national transmission. In addition to identifying high-priority districts for intervention, our results also indicate capacity for index case detection and outbreak response is most critical in the summer and during El Niño events.

## Conclusions

We present a three-stage approach to model the distribution of dengue outbreak vulnerability in Perú, facilitating tiered deployment of measures to prevent and mitigate outbreaks in both space and time. Our results demonstrate high validity of stage 1 (index case potential) and stage 2 (outbreak receptivity) scores for predicting outbreaks, and identify Selva Baja and Costa to be most vulnerable regions to dengue outbreaks, and summer and El Niño events to be the most vulnerable periods. Moving beyond the theoretical validity of these models and maps, we have conducted workshops and focus groups with representatives of health and environmental agencies in Perú to test their usability; we will present these results in a separate publication.

While we used dengue surveillance data to demonstrate the validity of our staged vulnerability model, their construction reflects factors common to outbreaks of other *Ae. ageypti*-vectored diseases in Perú, including Zika, yellow fever, and chikungunya. Namely, stage 1 and stage 2 vulnerability scores hold utility for predicting outbreaks of *Ae. aegypti*-vectored diseases, to which Selva Baja and Costa are particularly vulnerable. Furthermore, elements of stage 2 scores which reflect health systems strength are relevant to infectious disease outbreaks in general. Finally, successful extension of this framework from hemorrhagic fevers in Africa to *Ae. aeypti*-vectored diseases in Perú demonstrates its broad utility for outbreak and pandemic preparedness across settings and diseases.

## Supplementary information

**Additional file 1.** Vulnerability score maps stratified on El Niño and non-El Niño periods, winter (May–October).

**Additional file 2.** Vulnerability score maps stratified on El Niño and non-El Niño periods, summer (April–November).

**Additional file 3.** Hyperlink to visualization tool for vulnerability score maps, created using Tableau (University of Washington Tableau Public Server, https://public.tableau.com/en-us/s/).

**Additional file 4.** Seventeen figures presenting predictive model performance results for each stage (1–3) and season

## Data Availability

The dengue data used and/or analysed during the current study are available from Perú CDC (cvmunayco@gmail.com) on reasonable request. All other data were downloaded from publicly-available sources detailed in our References section.

## Abbreviations

EIP: Extrinsic incubation period
ENSO: El Niño Southern Oscillation
GLDAS: NASA’s Global Land Data Assimilation System
ICAR: Intrinsic conditional autoregressive model
ICEN: El Niño Coastal Index
SEIP: Probability of mosquito survival past the EIP
