# Supplementary material for: A novel approach to modeling epidemic vulnerability, applied to Aedes aegypti-vectored diseases in Perú
Source: BMC Infect Dis. 2021 Aug 21;21:846. doi: 10.1186/s12879-021-06530-9 (PMC8379593; doi:10.1186/s12879-021-06530-9)

Additional file 2: Vulnerability score maps stratified on El Niño and non-El Niño periods, summer (November - April)

Stage 1, Summer, El Nino

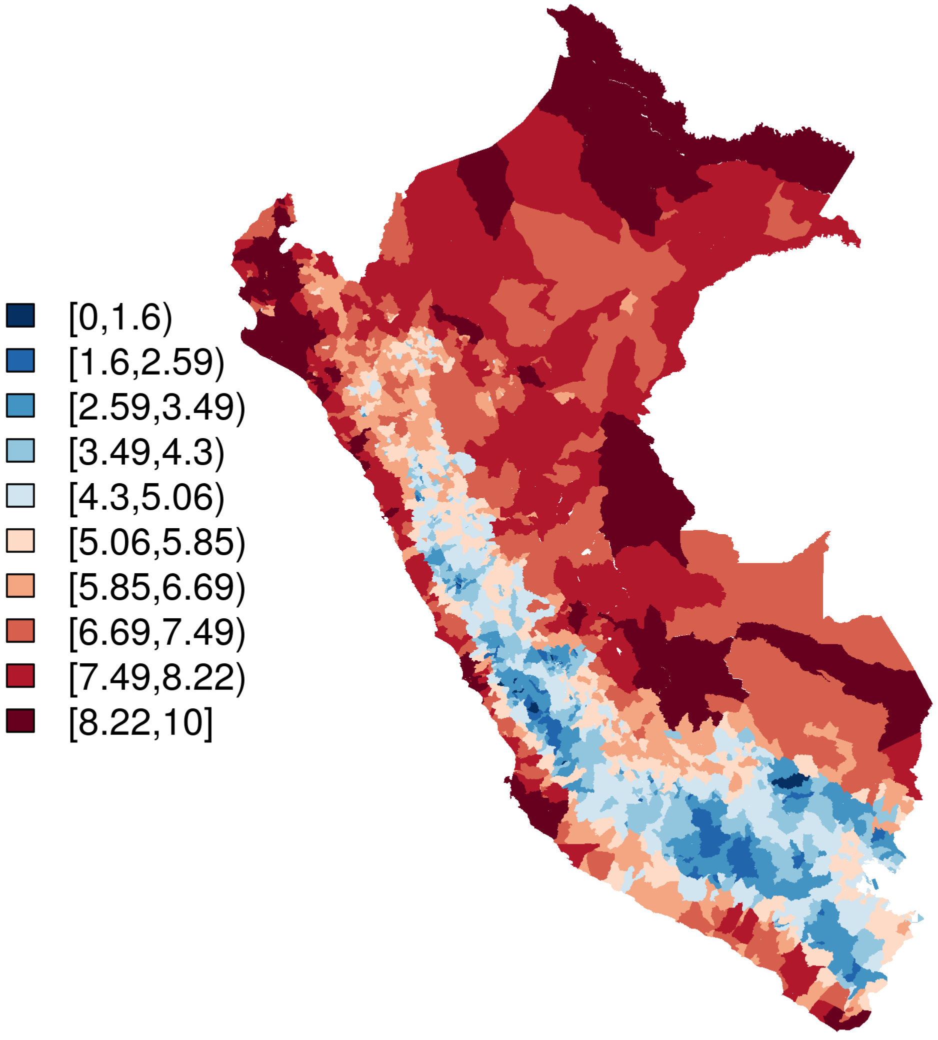

Stage 2, Summer, El Nino

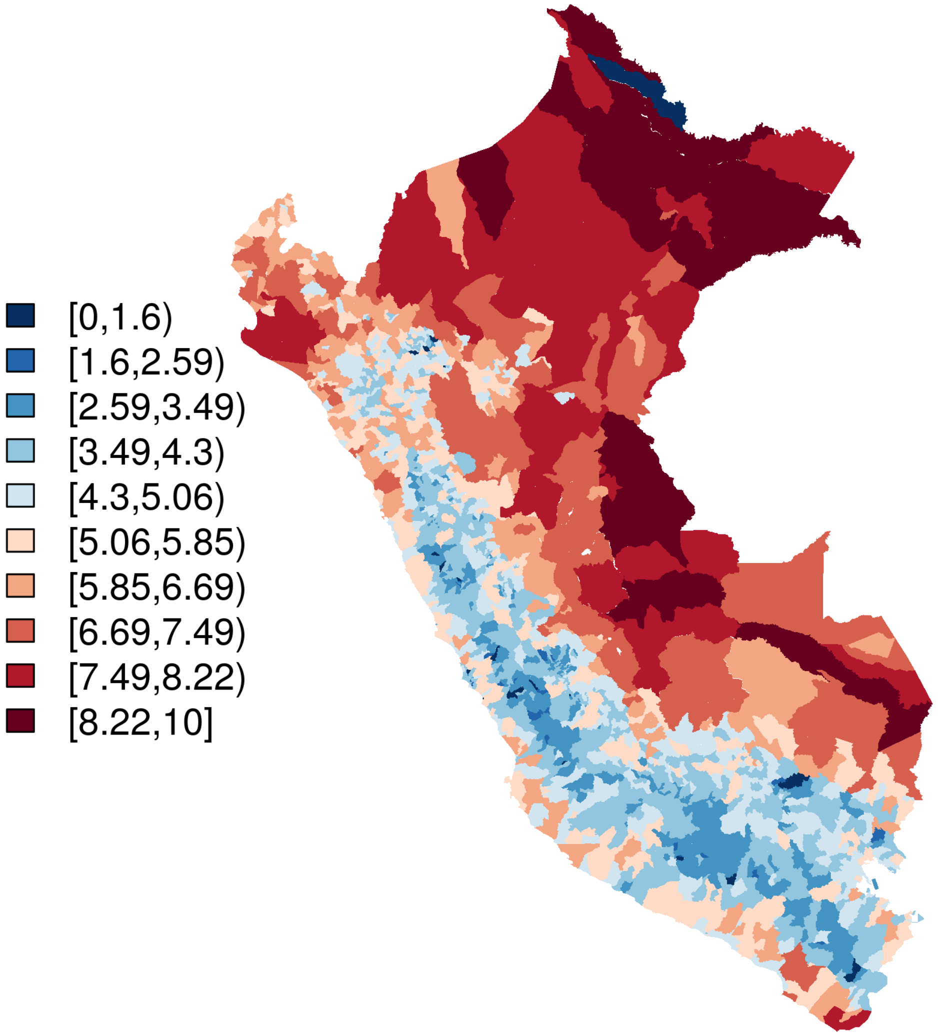

Stage 3, Summer, El Nino

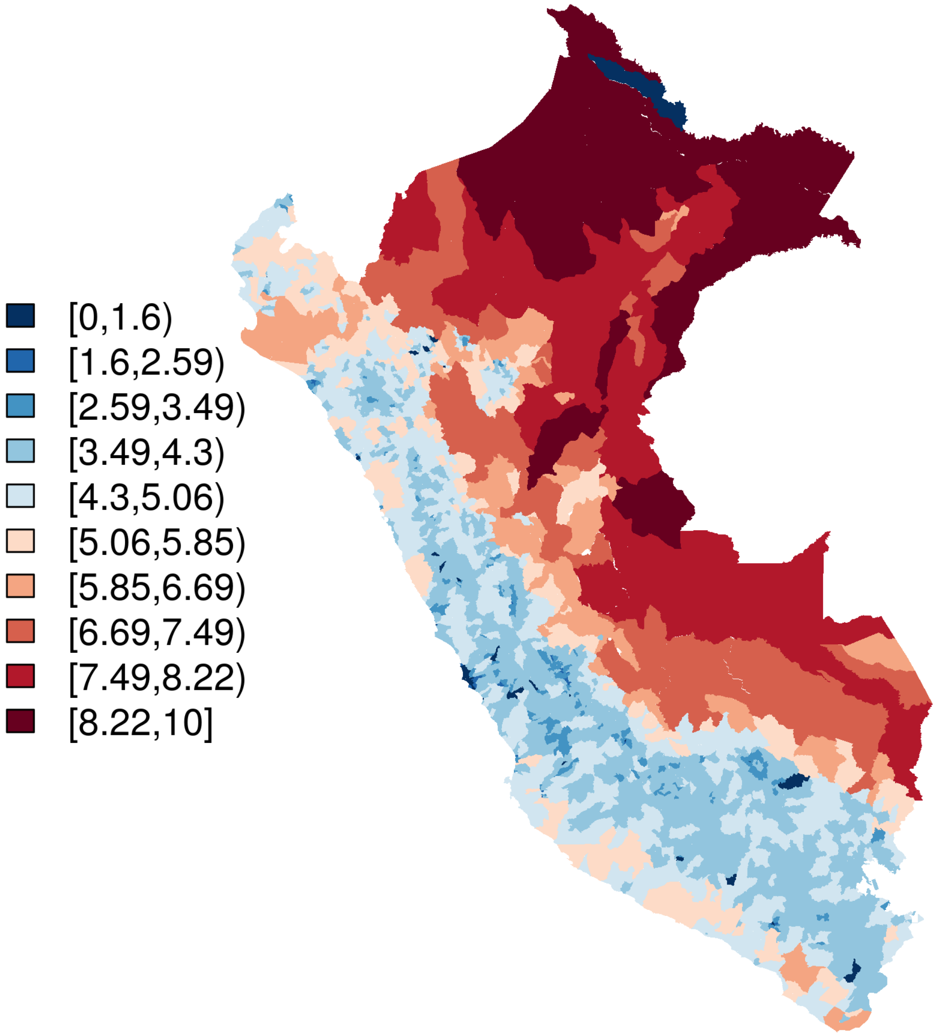

Stage 1, Summer, non-El Nino

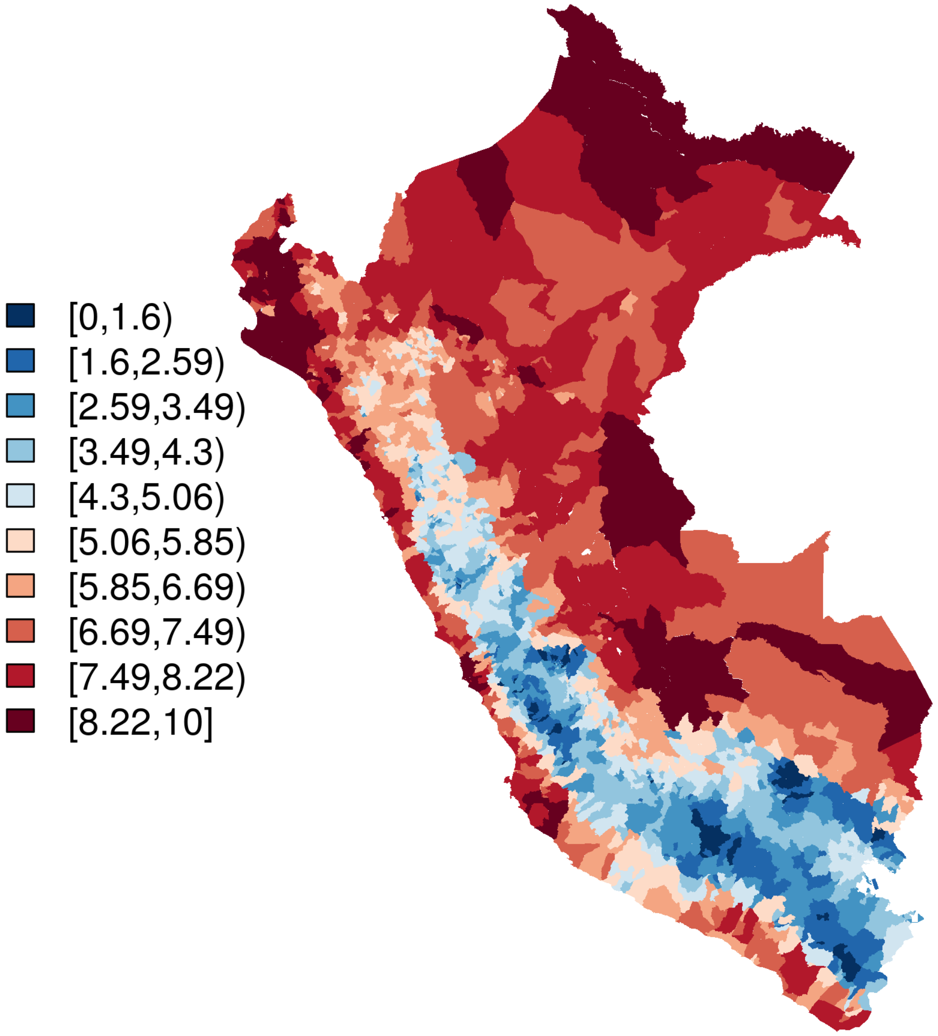

Stage 2, Summer, non-El Nino

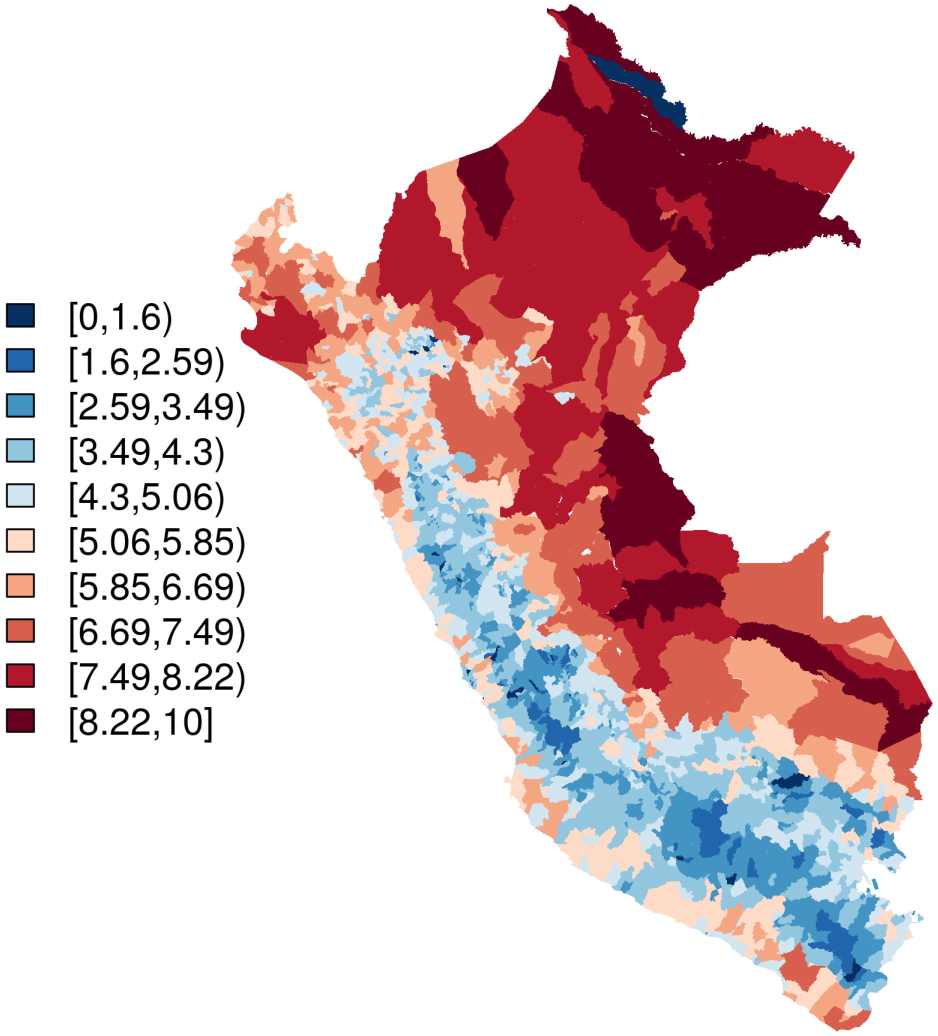

Stage 3, Summer, non-El Nino

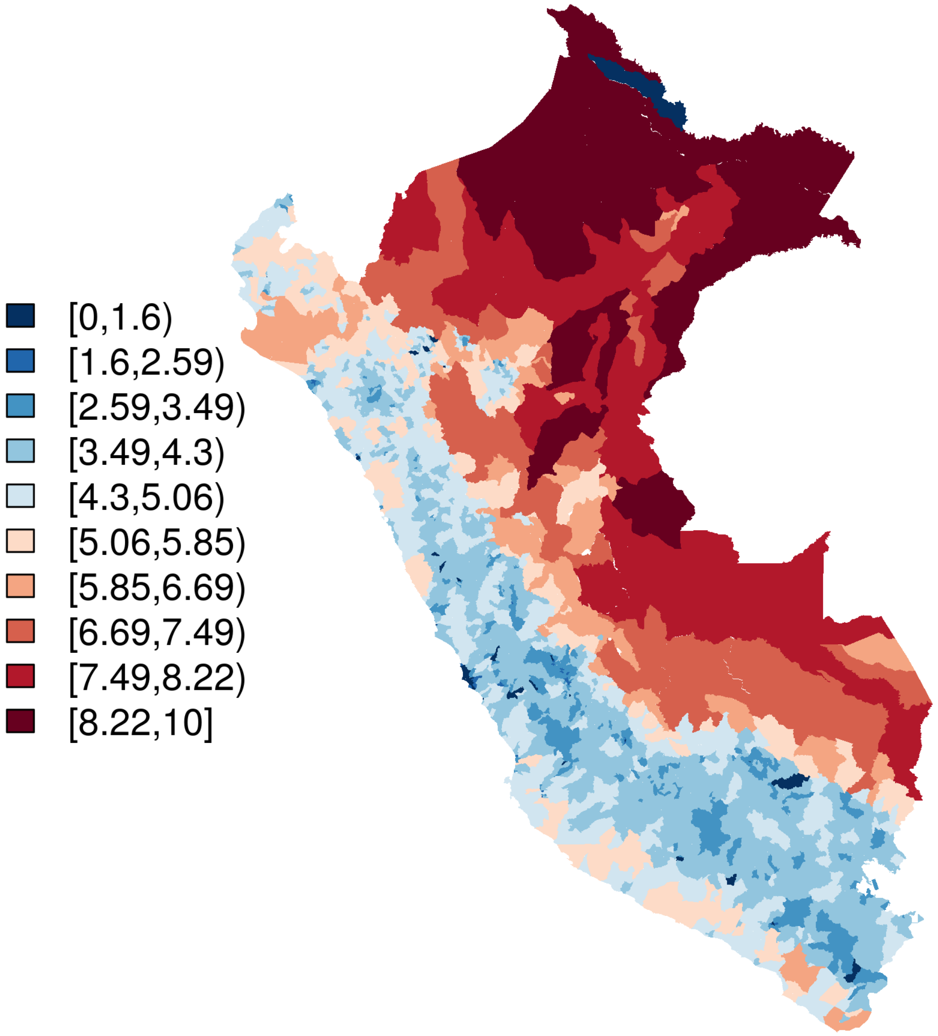

Supplement: Supplementary file 2 — Additional file 2. Vulnerability score maps stratified on El Niño and non-El Niño periods, summer (April–November). [file 12879_2021_6530_MOESM2_ESM.pdf]
