# Supplementary material for: A novel approach to modeling epidemic vulnerability, applied to Aedes aegypti-vectored diseases in Perú
Source: BMC Infect Dis. 2021 Aug 21;21:846. doi: 10.1186/s12879-021-06530-9 (PMC8379593; doi:10.1186/s12879-021-06530-9)
Supplement: Supplementary file 3 — Additional file 3. Hyperlink to visualization tool for vulnerability score maps, created using Tableau (University of Washington Tableau Public Server, https://public.tableau.com/en-us/s/). [file 12879_2021_6530_MOESM3_ESM.pdf]

S1 Link: Visualization Tool for Dengue Vulnerability Mapping in Peru

https://tableau.washington.edu/views/Maps-English/Story1?iframeSizedToWindow=true&:embed=y&:showAppBanner=false&:display_count=no&:showVizHome=no
