# Supplementary material for: A novel approach to modeling epidemic vulnerability, applied to Aedes aegypti-vectored diseases in Perú
Source: BMC Infect Dis. 2021 Aug 21;21:846. doi: 10.1186/s12879-021-06530-9 (PMC8379593; doi:10.1186/s12879-021-06530-9)
Supplement: Supplementary file 4 — Additional file 4. Seventeen figures presenting predictive model performance results for each stage (1–3) and season [file 12879_2021_6530_MOESM4_ESM.pdf]

Additional file 4: ROC curves

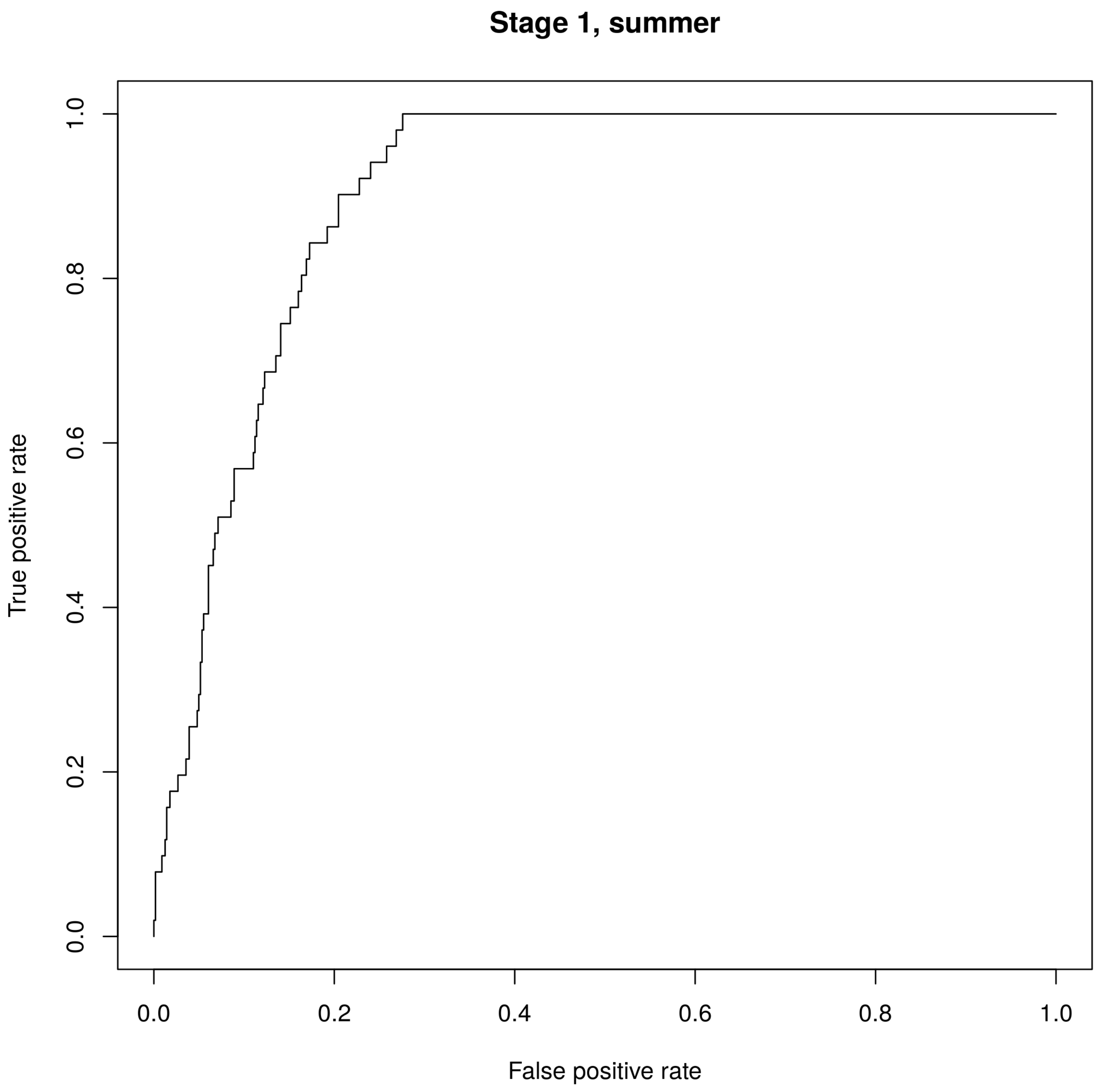

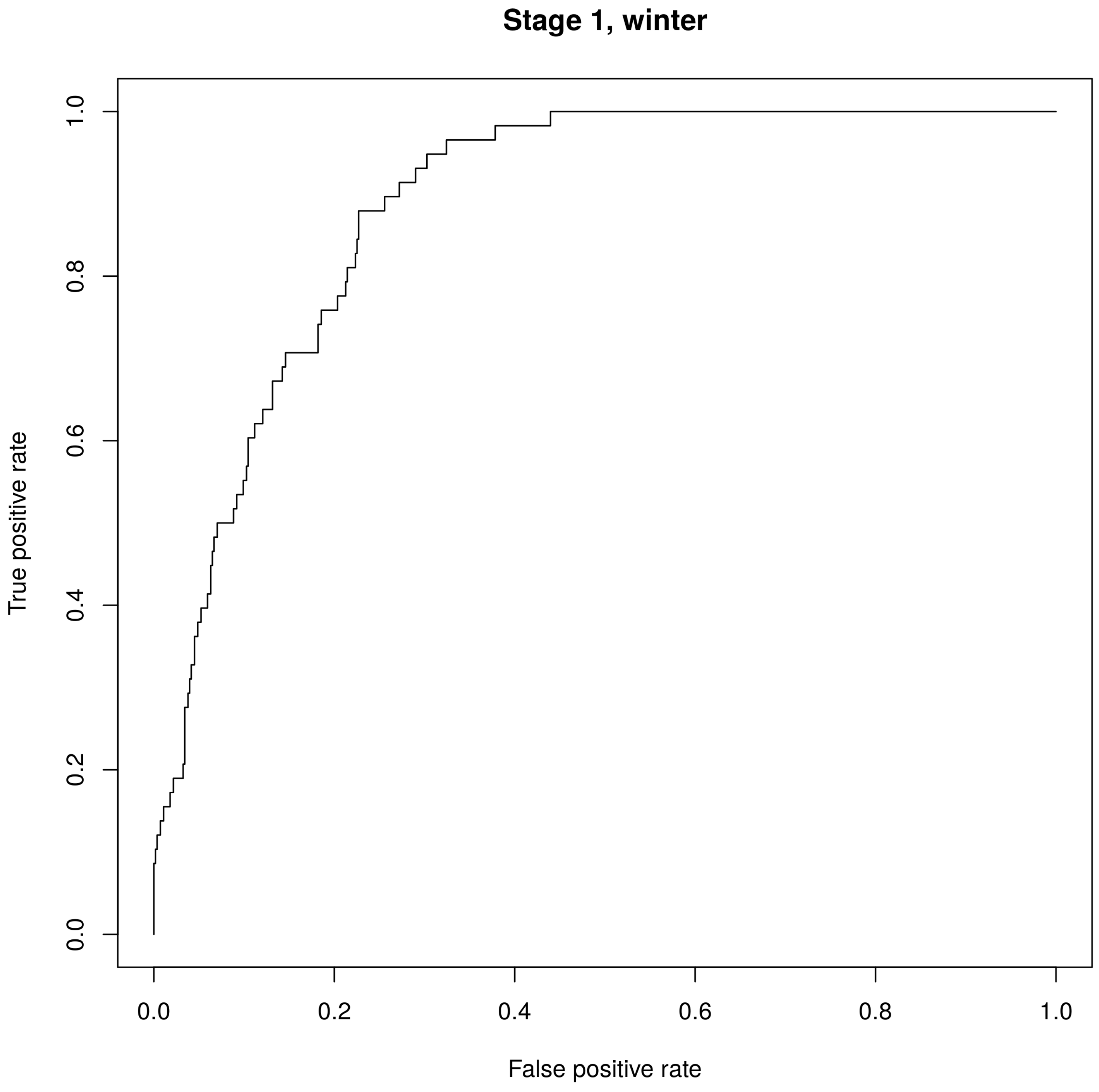

Stage 1, winter
True positive rate
False positive rate
0.0
0.2
0.4
0.6
0.8
1.0

Stage 1, summer, El Niño

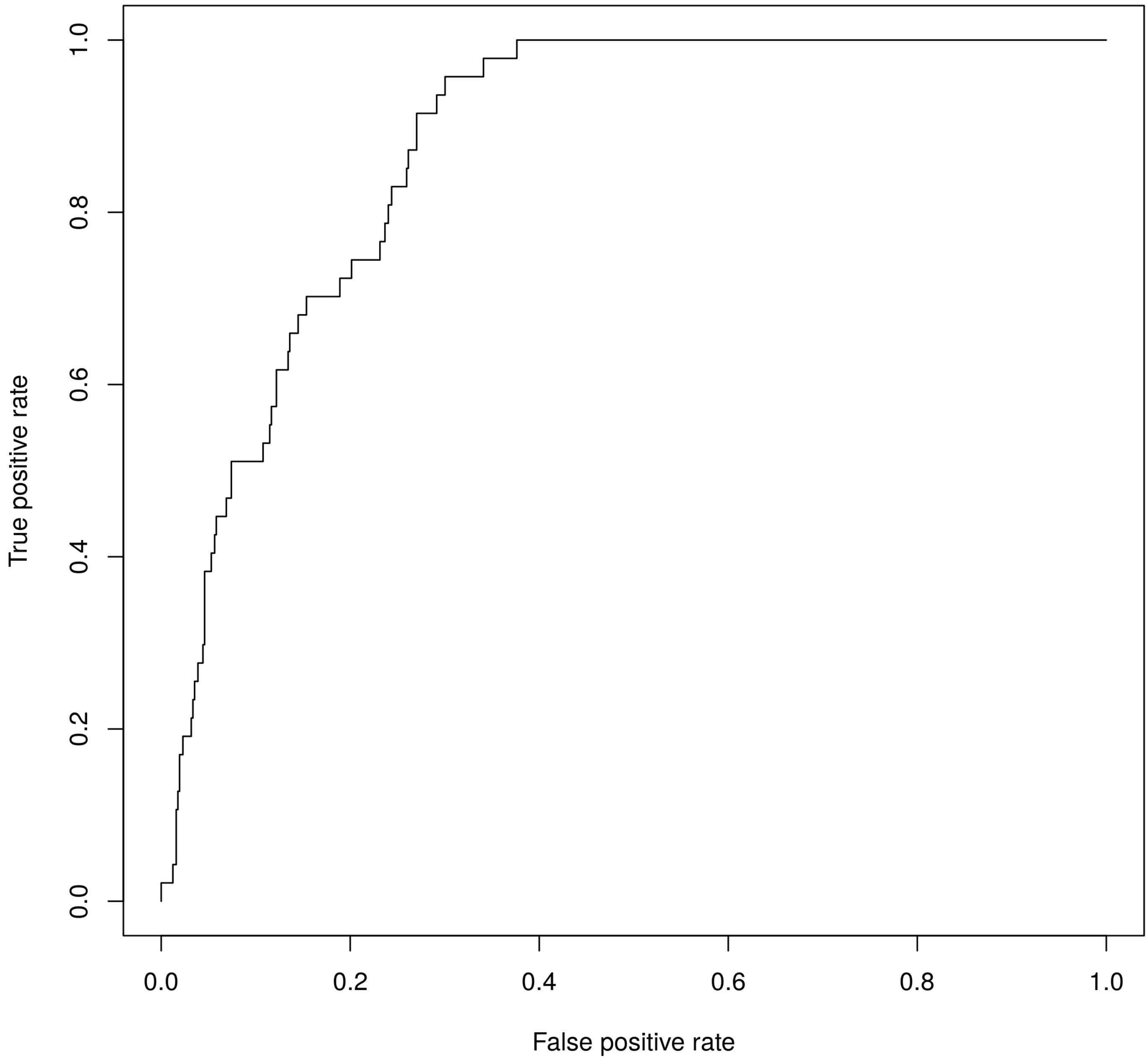

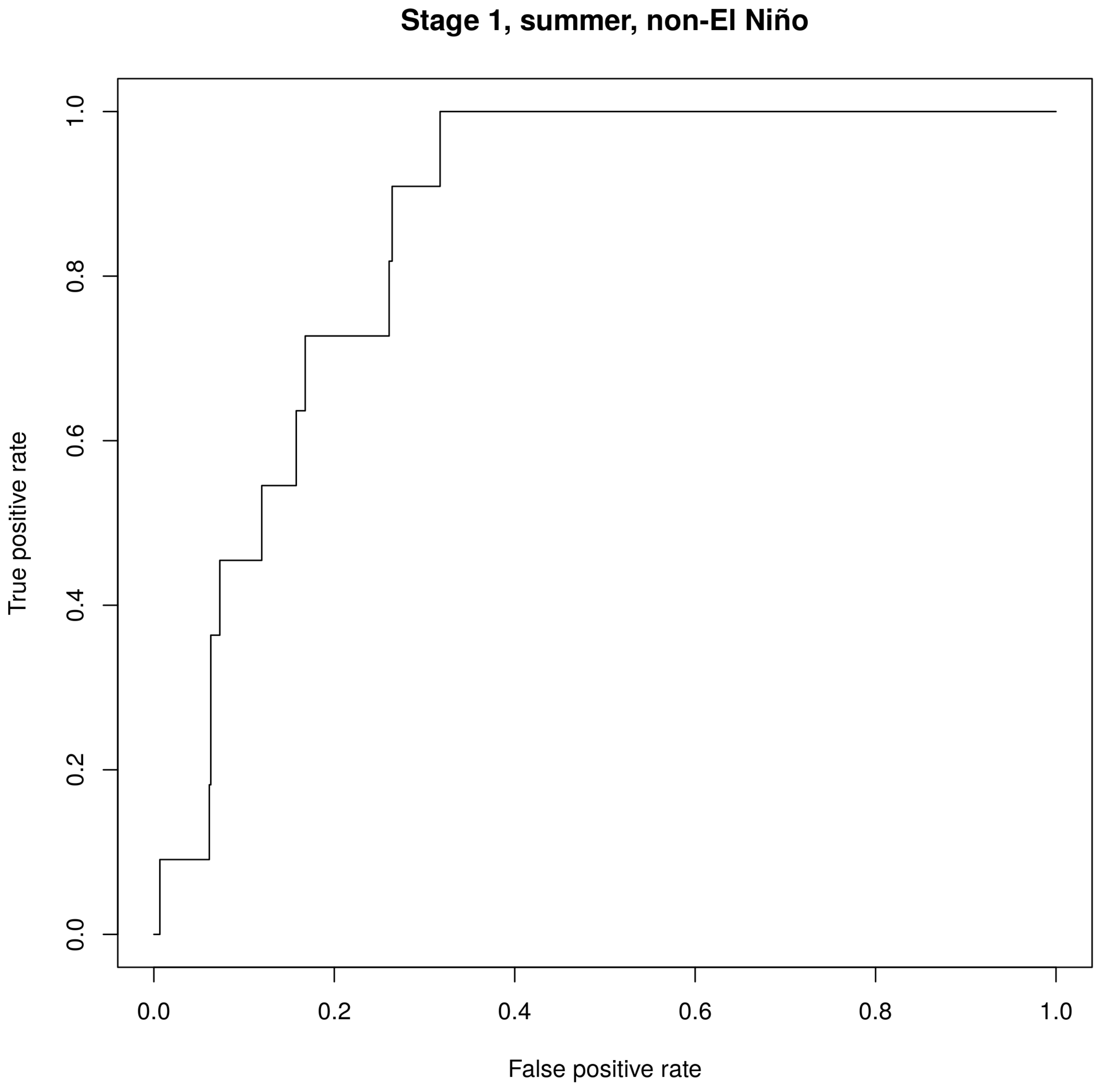
Stage 1, summer, non-El Niño
True positive rate
False positive rate
0.0
0.2
0.4
0.6
0.8
1.0

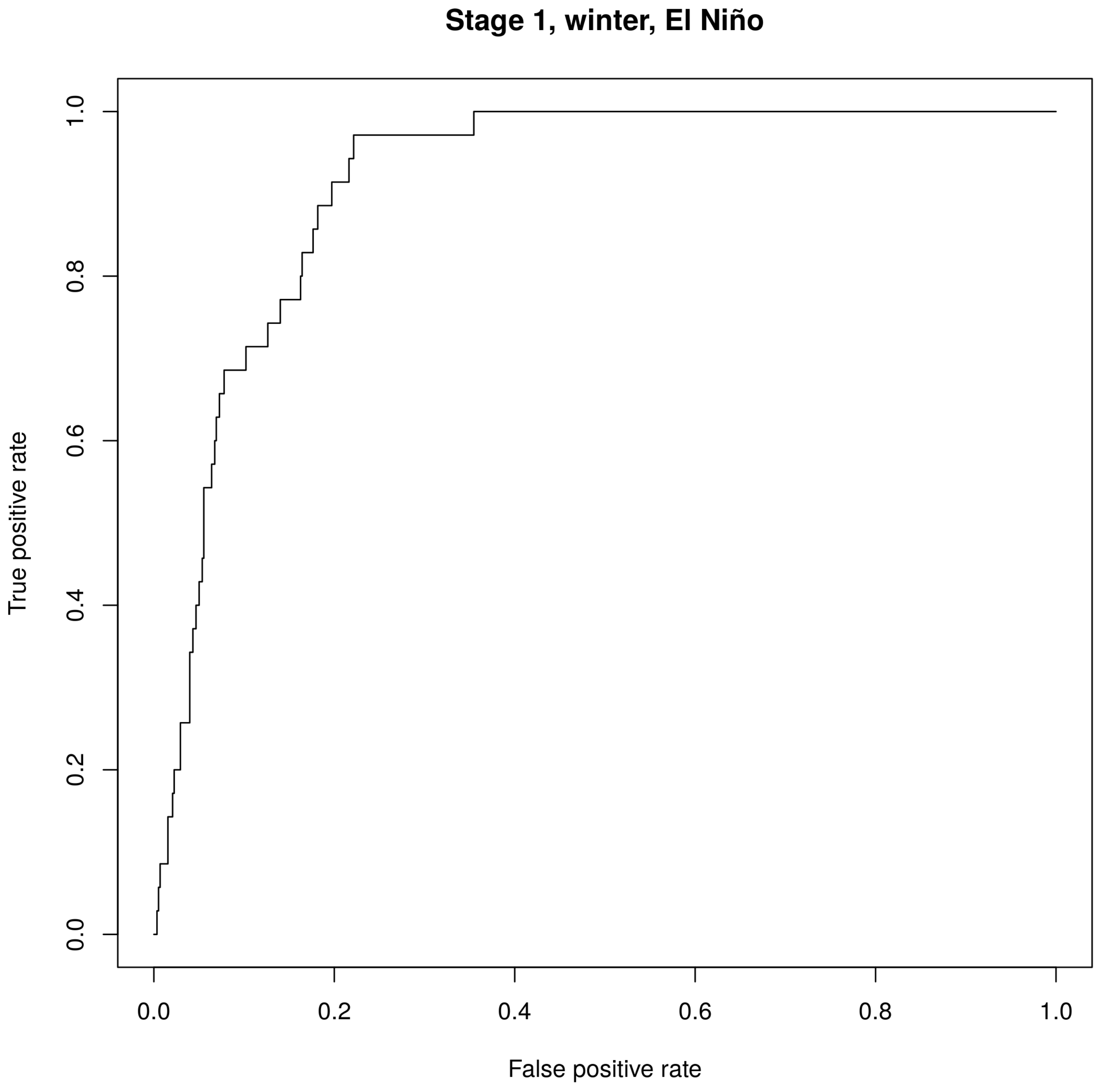
Stage 1, winter, El Niño
True positive rate
False positive rate
0.0
0.2
0.4
0.6
0.8
1.0

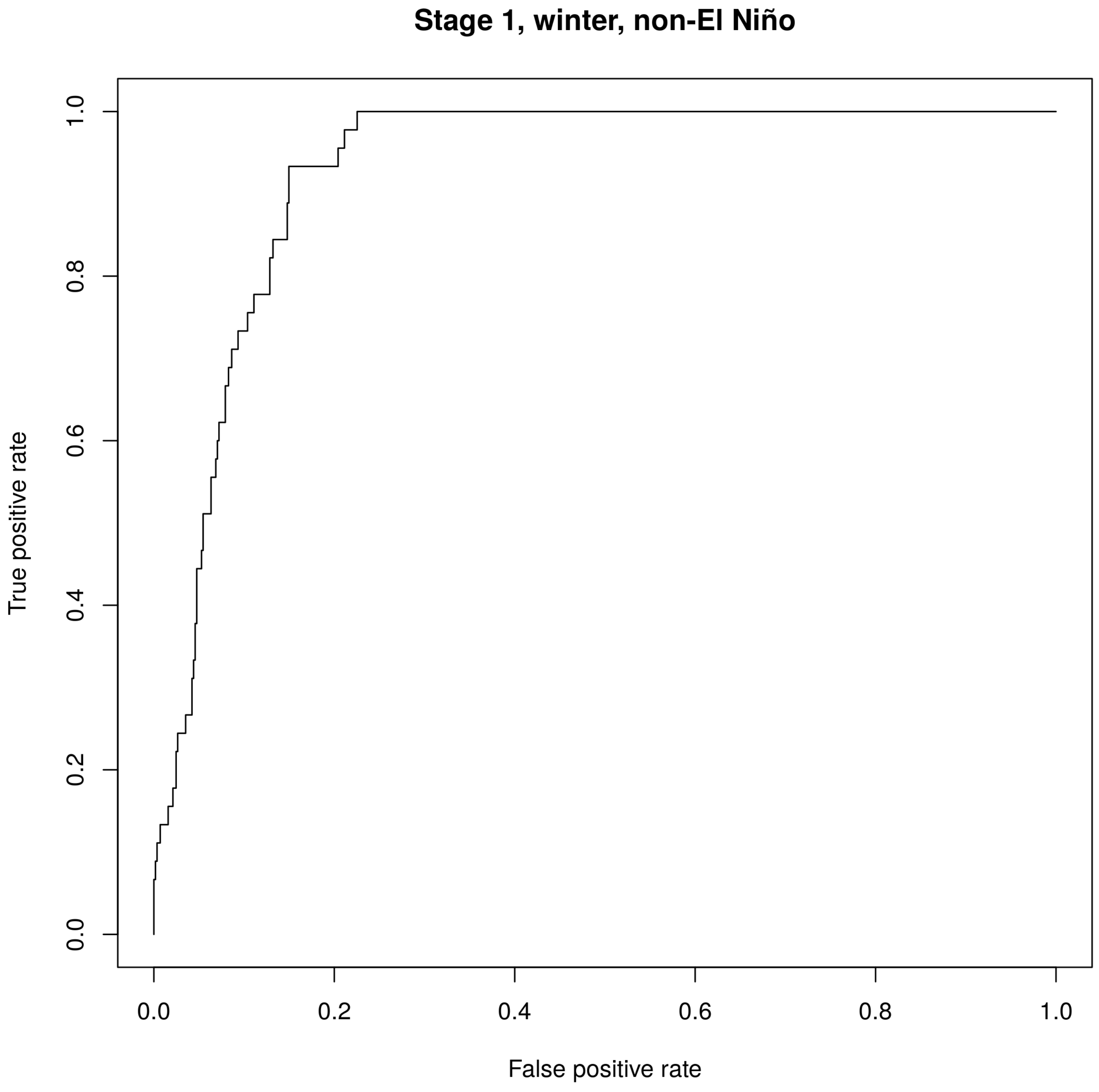

Stage 1, winter, non-El Niño
True positive rate
0.0
0.2
0.4
0.6
0.8
1.0
False positive rate
0.0
0.2
0.4
0.6
0.8
1.0

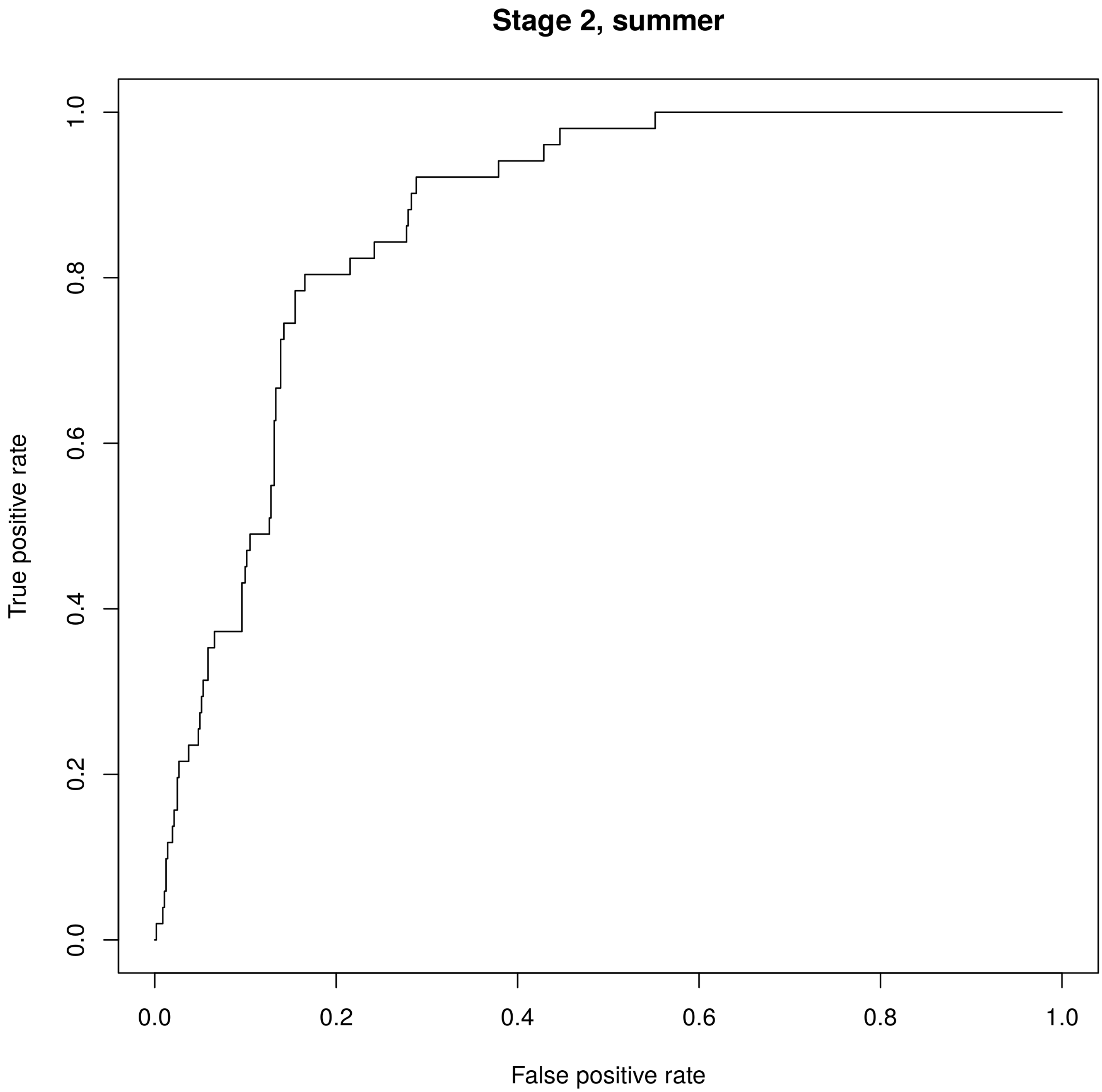
Stage 2, summer
True positive rate
False positive rate
0.0
0.2
0.4
0.6
0.8
1.0
0.0
0.2
0.4
0.6
0.8
1.0

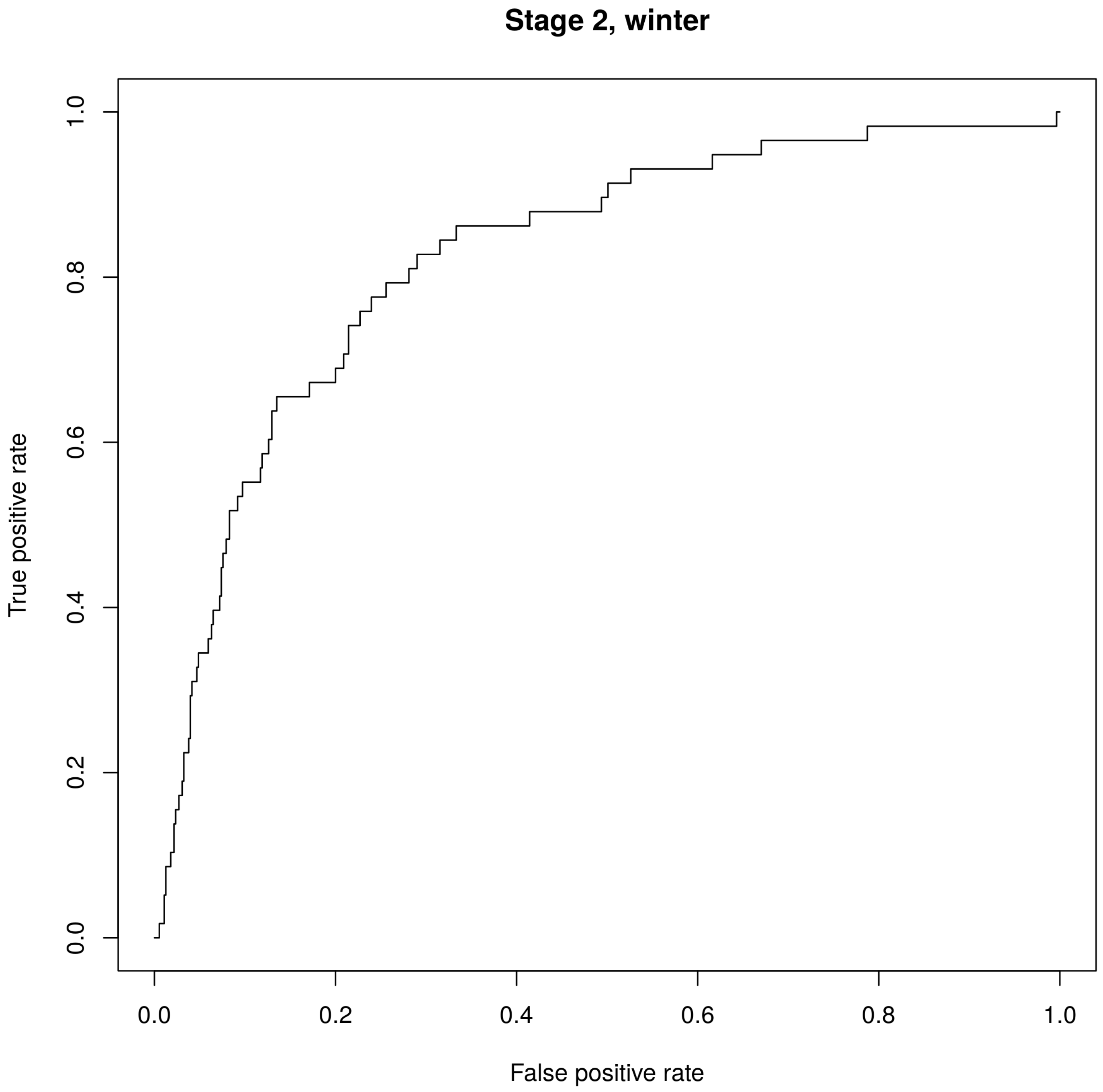

Stage 2, winter
True positive rate
0.0
0.2
0.4
0.6
0.8
1.0
False positive rate
0.0
0.2
0.4
0.6
0.8
1.0

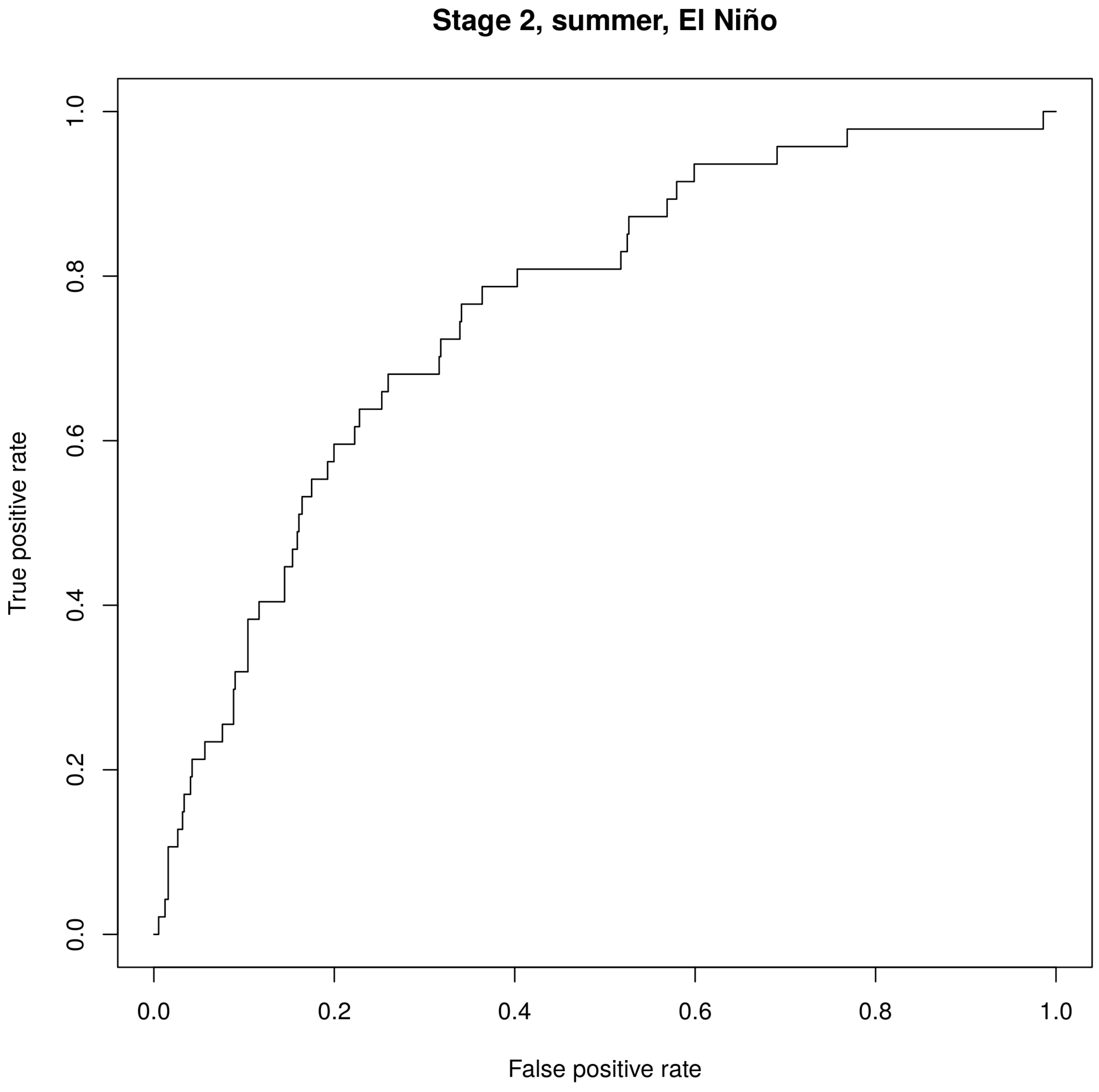
Stage 2, summer, El Niño
True positive rate
0.0
0.2
0.4
0.6
0.8
1.0
False positive rate
0.0
0.2
0.4
0.6
0.8
1.0

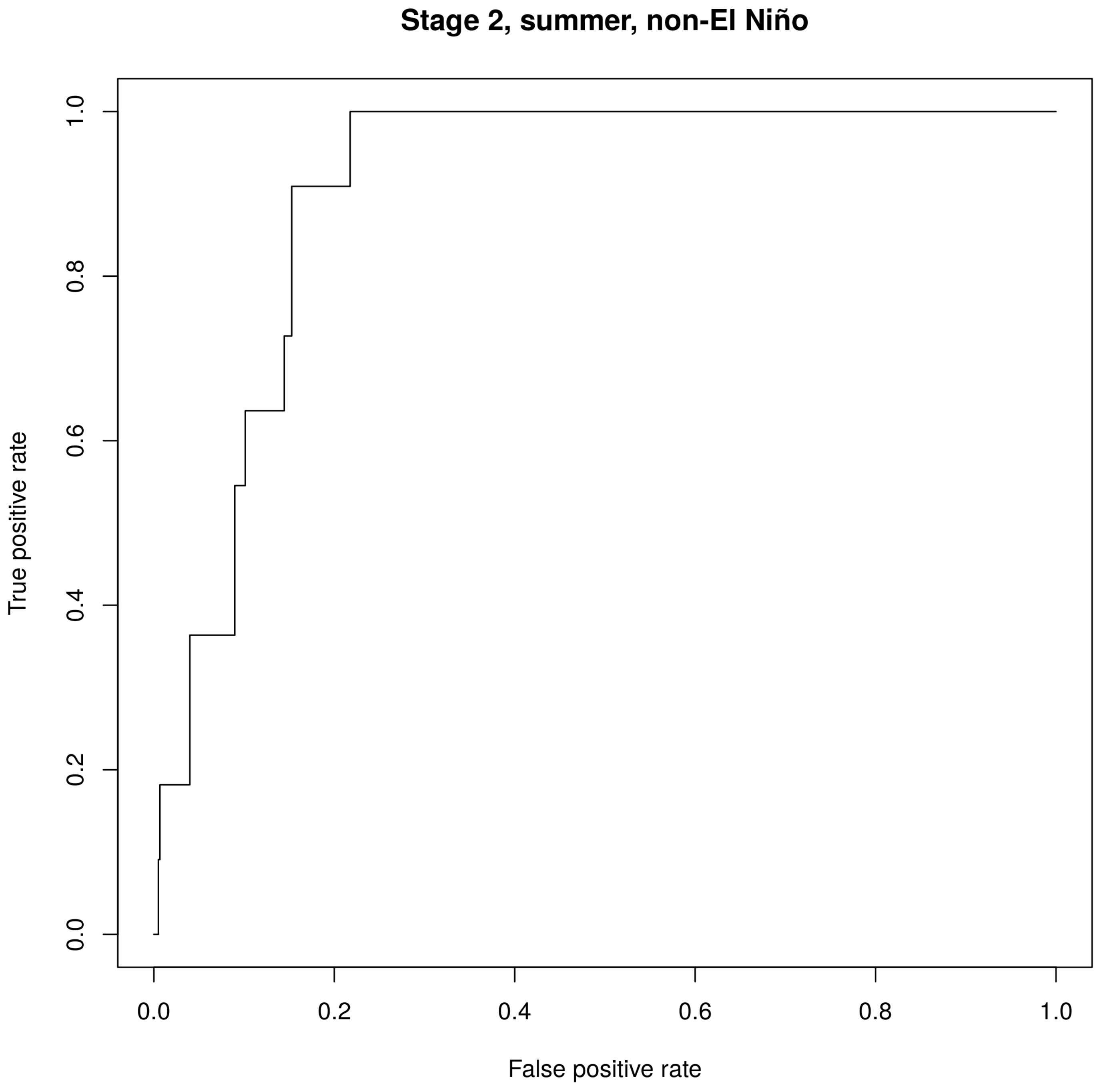
Stage 2, summer, non-El Niño
True positive rate
False positive rate
0.0
0.2
0.4
0.6
0.8
1.0

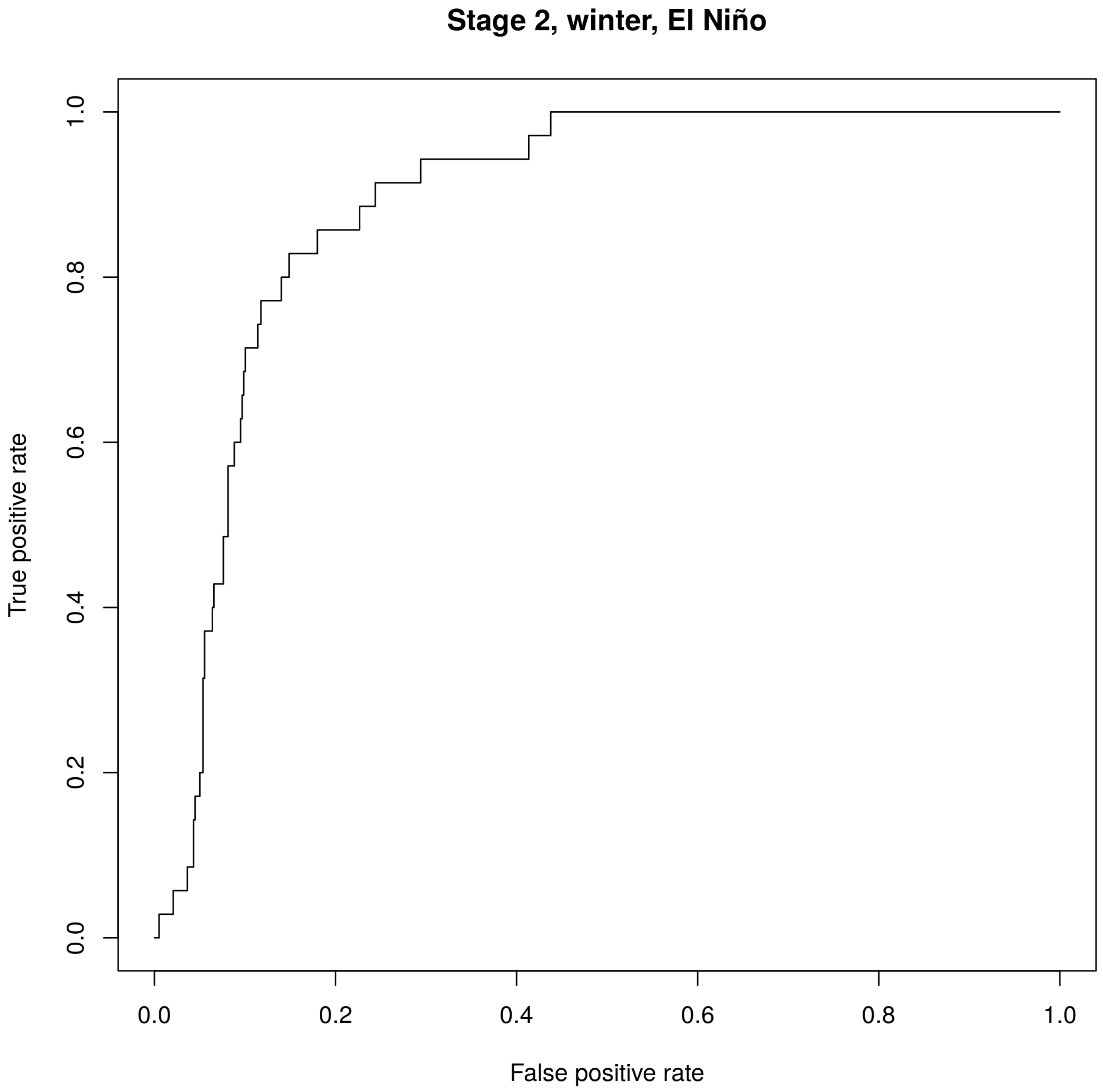

Stage 2, winter, El Niño
True positive rate
0.0
0.2
0.4
0.6
0.8
1.0
0.0
0.2
0.4
0.6
0.8
1.0
False positive rate

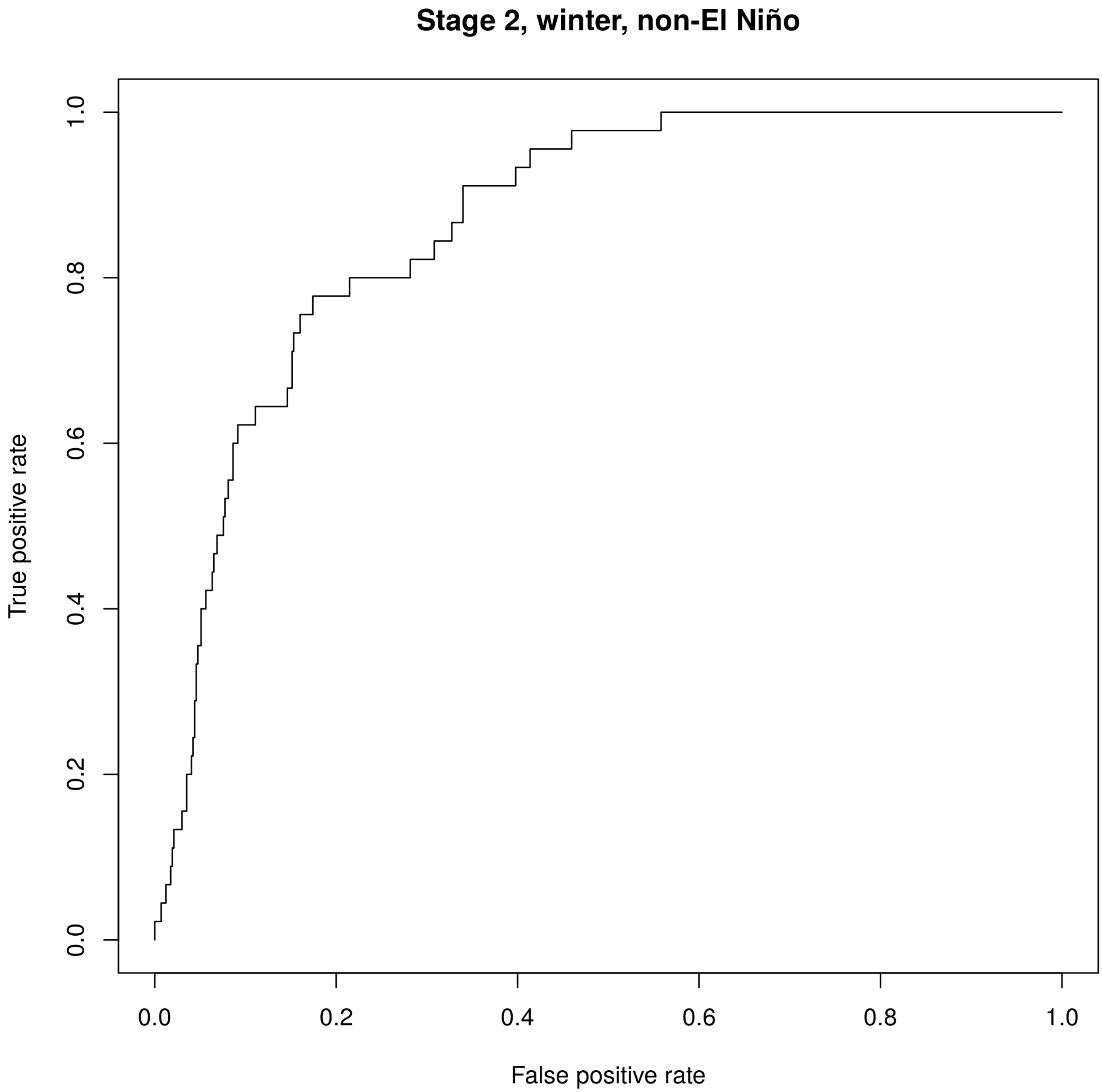
Stage 2, winter, non-El Niño
True positive rate
False positive rate
0.0
0.2
0.4
0.6
0.8
1.0

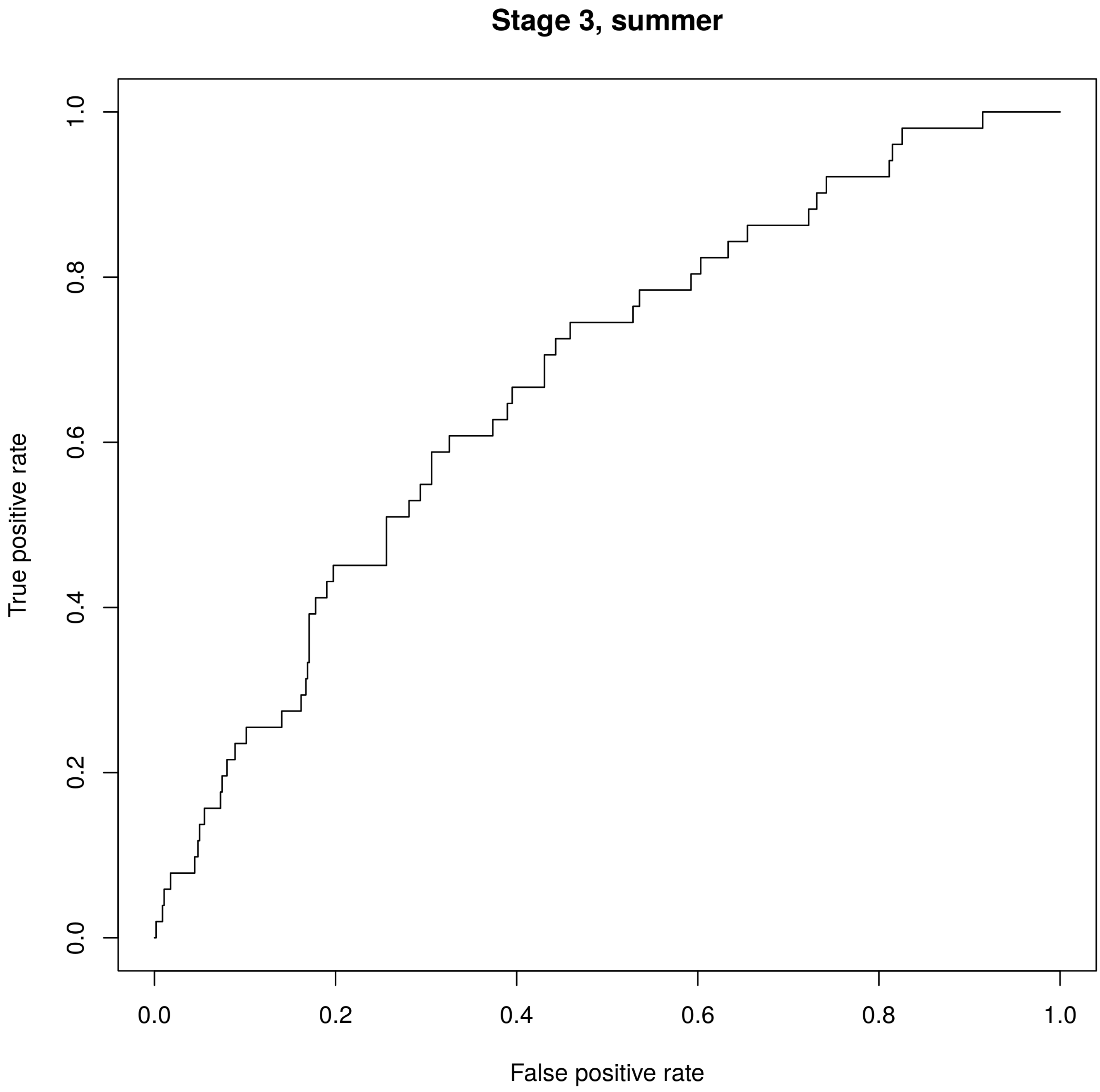

Stage 3, summer
True positive rate
False positive rate
0.0
0.2
0.4
0.6
0.8
1.0

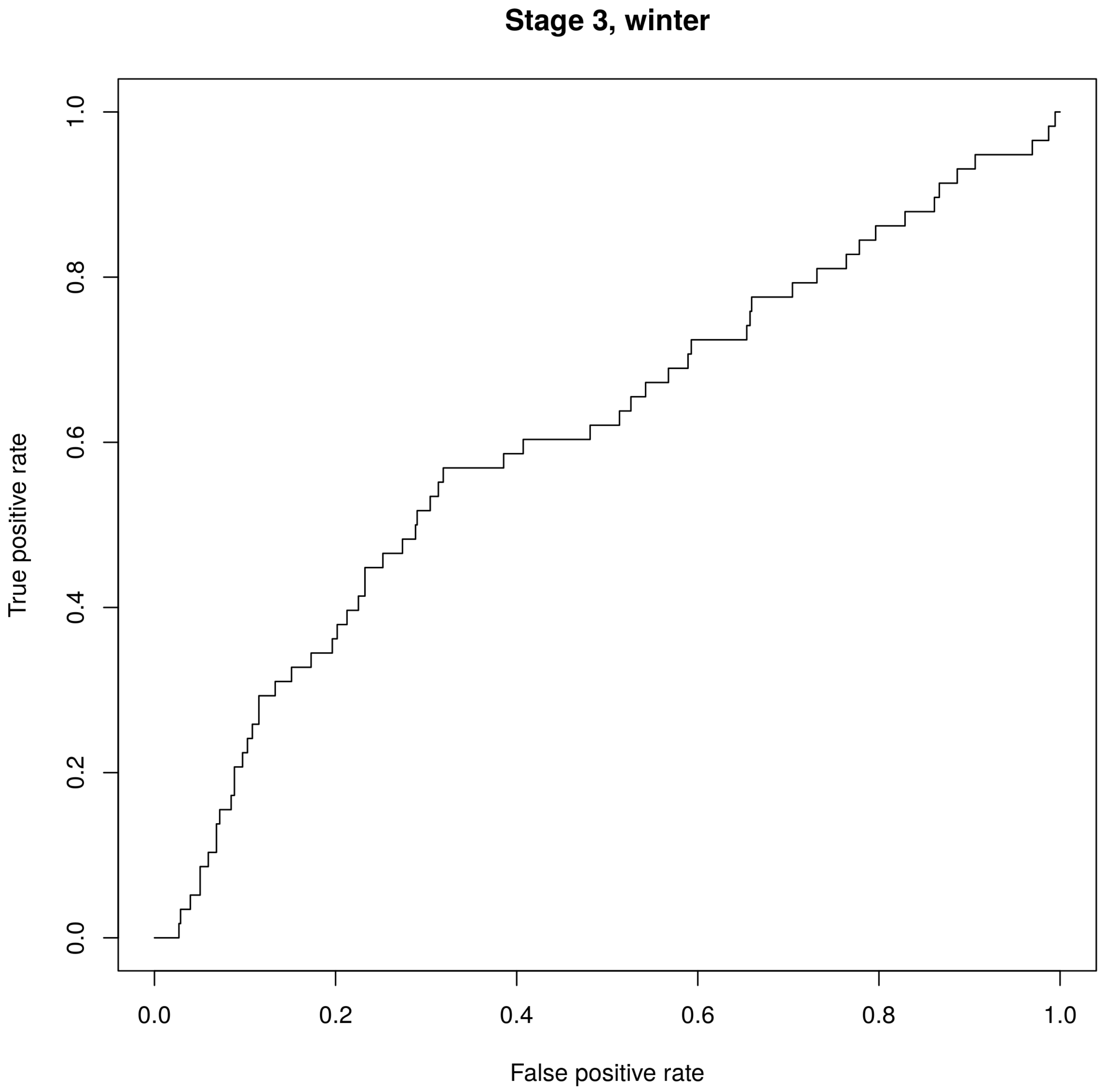
Stage 3, winter
True positive rate
0.0
0.2
0.4
0.6
0.8
1.0
0.0
0.2
0.4
0.6
0.8
1.0
False positive rate

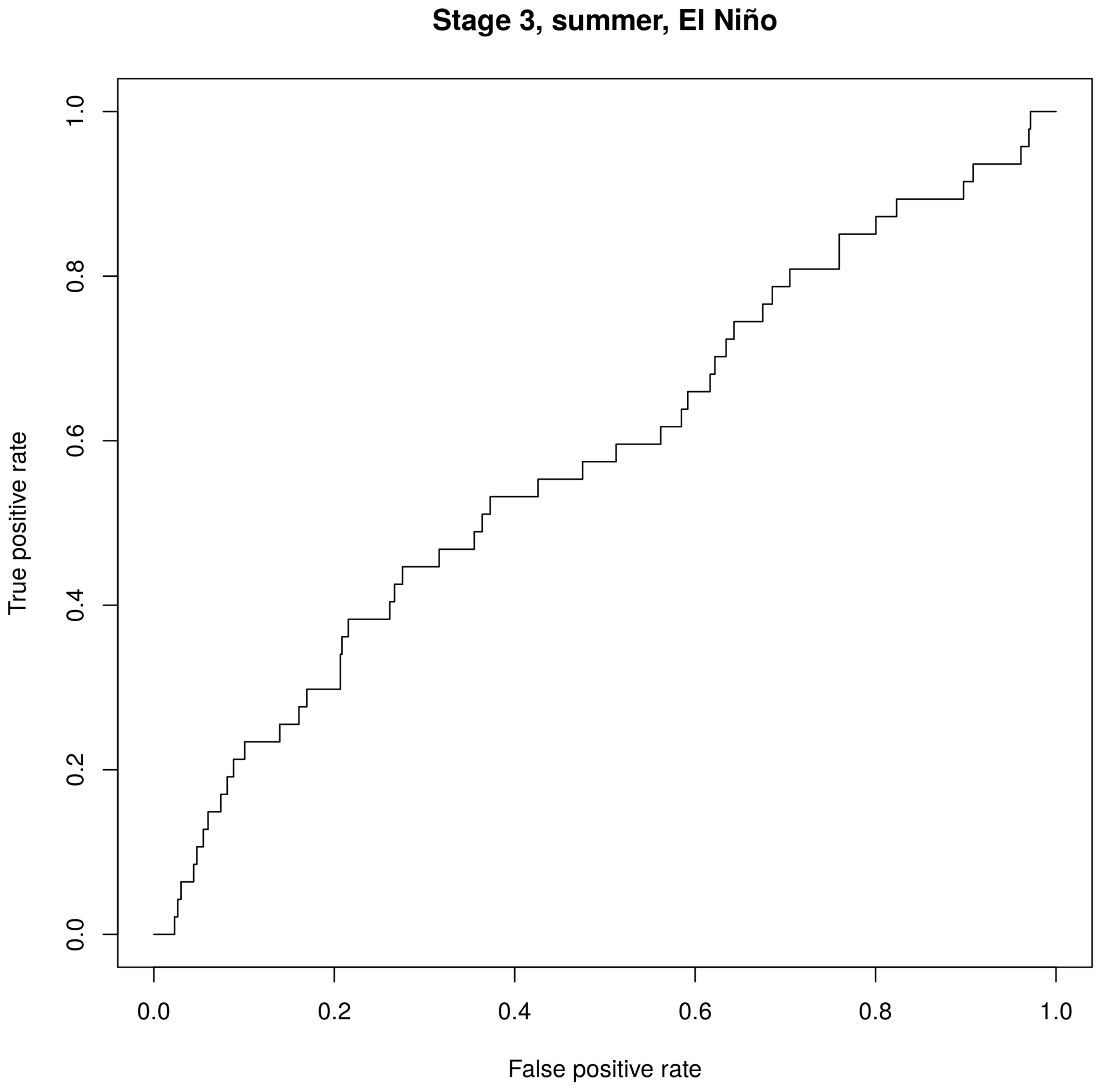
Stage 3, summer, El Niño
True positive rate
False positive rate
0.0
0.2
0.4
0.6
0.8
1.0
0.0
0.2
0.4
0.6
0.8
1.0

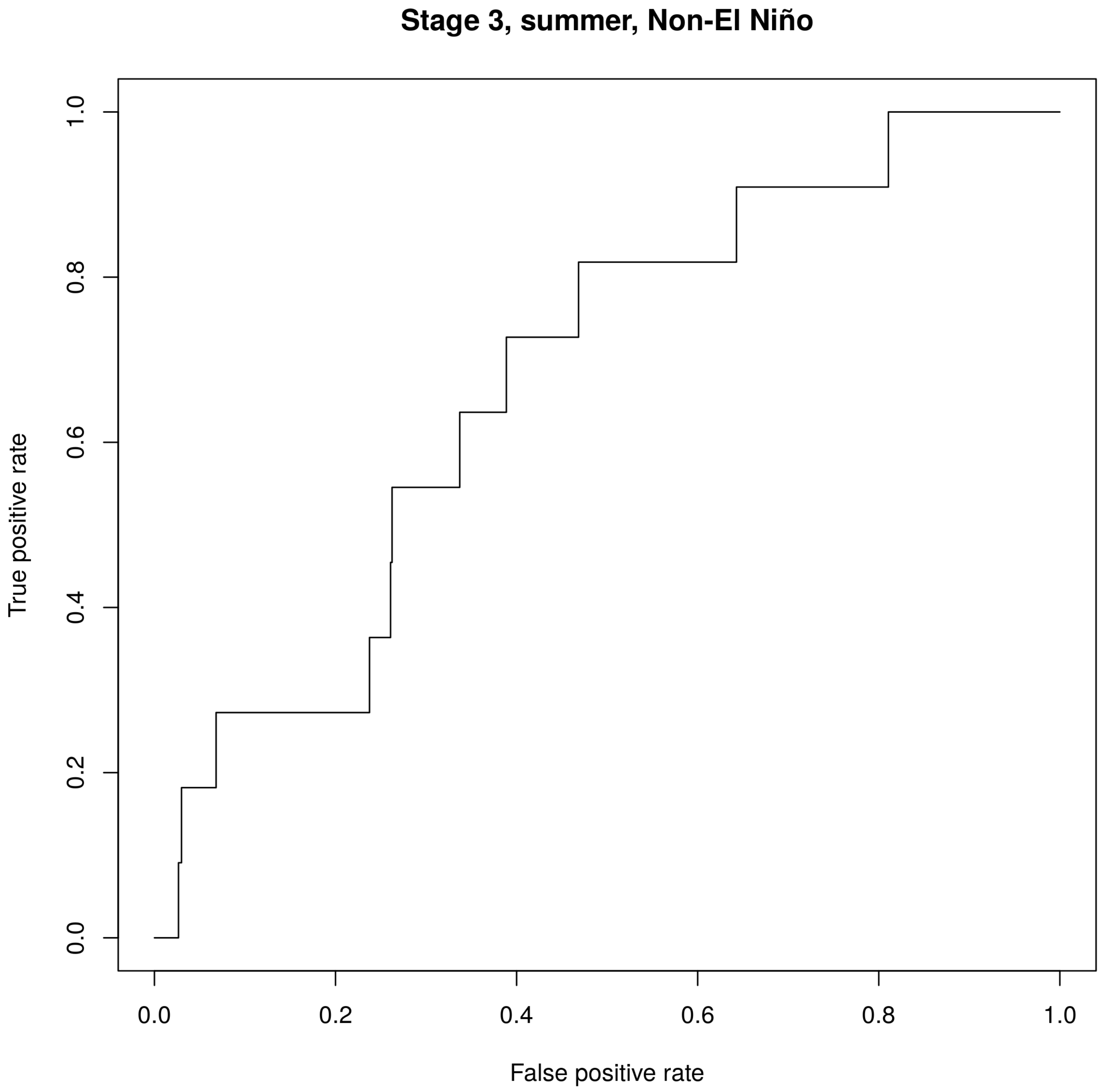
Stage 3, summer, Non-El Niño
True positive rate
False positive rate
0.0
0.2
0.4
0.6
0.8
1.0

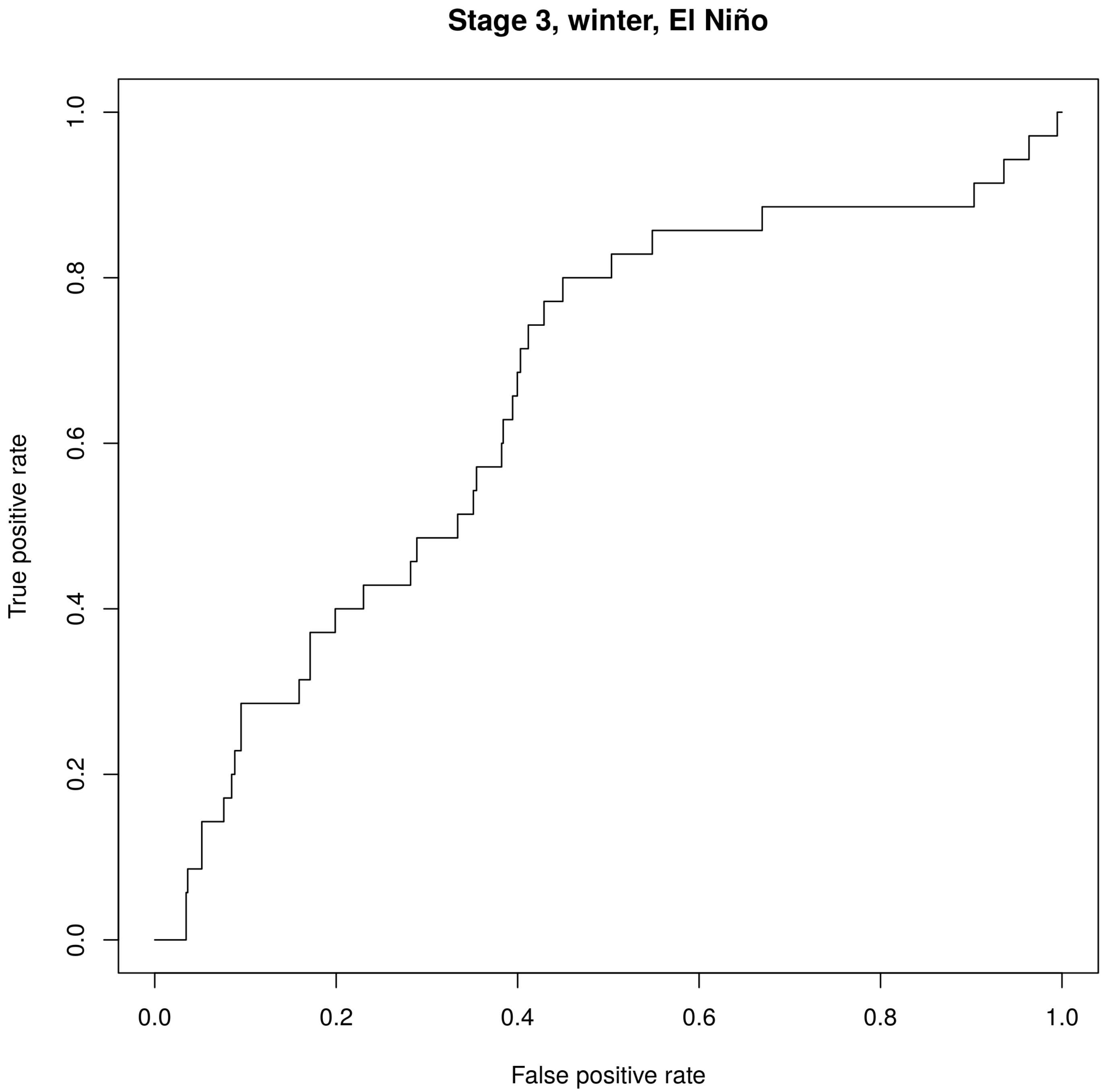

Stage 3, winter, El Niño
True positive rate
0.0
0.2
0.4
0.6
0.8
1.0
False positive rate
0.0
0.2
0.4
0.6
0.8
1.0

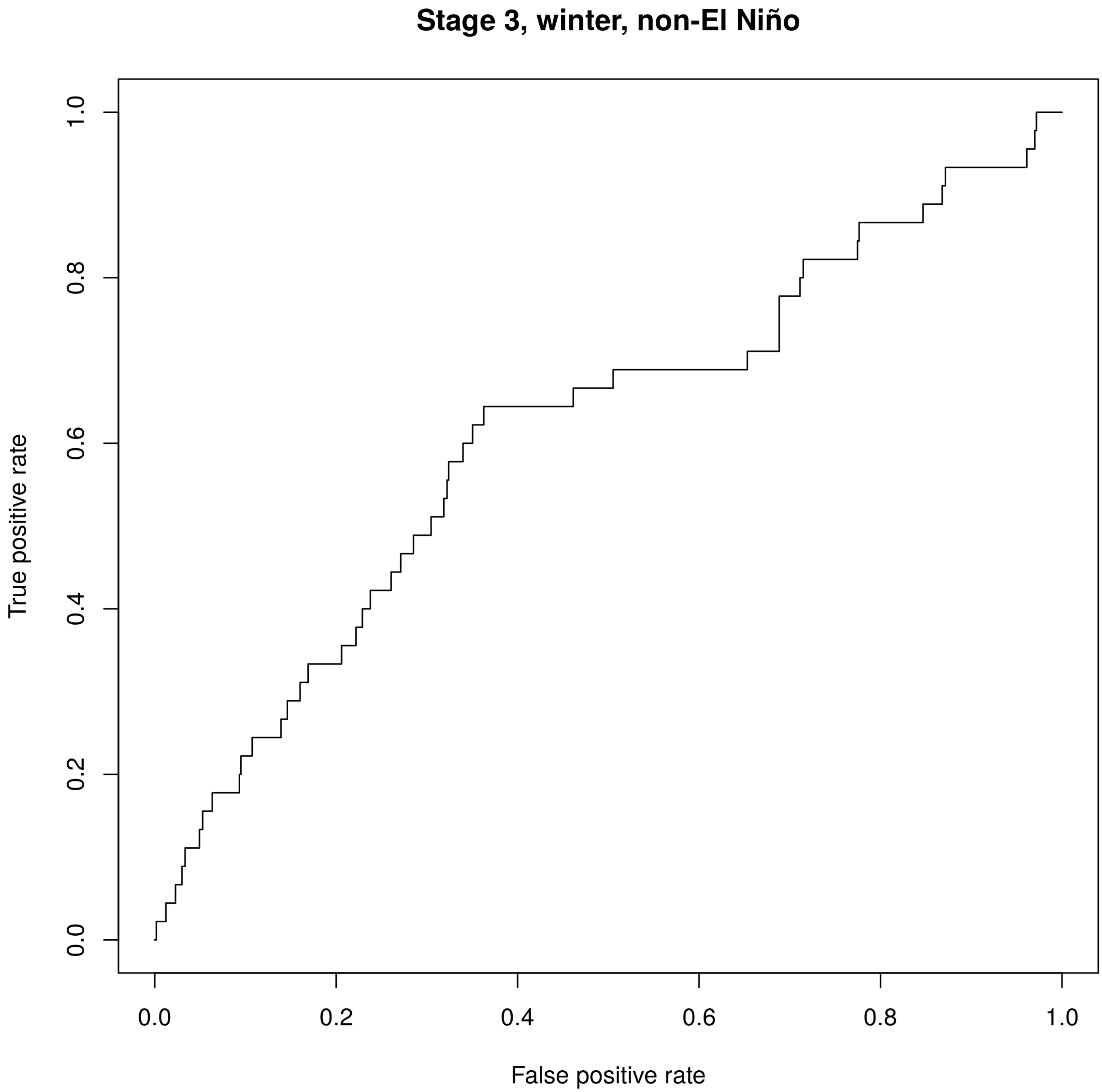
Stage 3, winter, non-El Niño
True positive rate
0.0
0.2
0.4
0.6
0.8
1.0
False positive rate
0.0
0.2
0.4
0.6
0.8
1.0
